# Cyclooxygenase-2/prostaglandin E2 pathway orchestrates the replication of infectious bronchitis virus in chicken tracheal explants

**DOI:** 10.1128/spectrum.00407-24

**Published:** 2024-10-29

**Authors:** Motamed Elsayed Mahmoud, Ahmed Ali, Muhammad Farooq, Ishara M. Isham, Sufna M. Suhail, Heshanthi Herath-Mudiyanselage, Ryan Rahimi, Mohamed Faizal Abdul-Careem

**Affiliations:** 1 Faculty of Veterinary Medicine, University of Calgary, Calgary, Alberta, Canada; 2 Department of Animal Husbandry, Faculty of Veterinary Medicine, Sohag University, Sohag, Egypt; 3 Department of Pathology, Faculty of Veterinary Medicine, Beni-Suef University, Beni Suef, Egypt; Wuhan Institute of Virology, Chinese Academy of Sciences, Wuhan, China

**Keywords:** infectious bronchitis virus, tracheal organ culture, cyclooxygenase 2, prostaglandin E2, selective cyclooxygenase-2 inhibitor, prostaglandin receptor antagonists, interferon-γ, Janus-kinase inhibitors, interleukins

## Abstract

In this study, we investigated the localized pathogenesis of infectious bronchitis virus (IBV) in chicken tracheal organ cultures (TOCs), focusing on the role of inducible cyclooxygenase (COX-2). Two divergent IBV strains, respiratory Connecticut (Conn) A5968 and nephropathogenic Delmarva (DMV)/1639, were studied at 6, 12, 24, and 48 hours post-infection (hpi). Various treatments including exogenous prostaglandin (PGE)2, a selective COX-2 antagonist (SC-236), and inhibitors of PGE2 receptors and Janus kinase (JAK) were administered. IBV genome load and antigen expression were quantified using real-time quantitative PCR and immunohistochemistry. COX-2, interferon (IFN)-α, IFN-β, interleukin (IL)−1β, IL-6, and inducible nitric oxide synthase (iNOS) expressions were measured, along with PGE2 and COX-2 concentrations. IBV genome load and protein expression peaked at 12 and 24 hpi, respectively. Conn A5968-infected TOCs exhibited continuous COX-2 expression for up to 24 hpi, extended PGE2 production up to 48 hpi, and reduced inflammatory cytokine expression. In contrast, DMV/1639-infected TOCs displayed heightened inflammatory cytokine expression, brief COX-2 expression, and PGE2 production. Treatment with IFN-γ, SC-236, PGE2 receptor inhibitors, or JAK inhibitors reduced IBV infection and lesion scores, whereas exogenous PGE2 or IFN-γ pretreatment with a JAK-2 inhibitor augmented infection. These findings shed light on the innate immune regulation of IBV infection in the trachea, highlighting the involvement of the COX-2/PGE2 pathway.

**IMPORTANCE:**

Understanding the localized pathogenesis of infectious bronchitis virus (IBV) within the trachea of chickens is crucial for developing effective control strategies against this prevalent poultry pathogen. This study sheds light on the role of inducible cyclooxygenase (COX-2) and prostaglandin (PGE)2 in IBV pathogenesis using chicken tracheal organ culture (TOC) models. The findings reveal distinct patterns of COX-2 expression, PGE2 production, and immune responses associated with different IBV strains, highlighting the complexity of host-virus interactions. Furthermore, the identification of specific inhibitors targeting the COX-2/PGE2 pathway and Janus kinase-signal transducer and activator of transcription (JAK-STAT) signaling pathway provides potential therapeutic avenues for mitigating IBV infection in poultry. Overall, this study contributes to our understanding of the innate immune regulation of IBV infection within the trachea, laying the groundwork for the development of targeted interventions to control IBV outbreaks in poultry populations.

## INTRODUCTION

Infectious bronchitis virus (IBV) is a widespread avian respiratory pathogen known for its wide-ranging tissue tropism, affecting various systems in chickens, including the respiratory tract, reproductive, gastrointestinal, and renal systems. It demonstrates a particular affinity for epithelial and lymphoid organs (1). The primary target of IBV is the respiratory epithelium, establishing itself as the main point of entry (2). Importantly, IBV showcases the ability to replicate in both hematopoietic and non-hematopoietic cells, allowing productive infections in monocytes and macrophages (3–5). Furthermore, certain IBV strains exhibit specific tropism, categorized as respiratory or nephropathogenic strains (6, 7). For instance, the IBV strain Delmarva (DMV/1639) is predominantly classified as nephropathogenic, causing severe kidney lesions and leading to significant renal pathology in infected chickens (8, 9). This strain’s unique pathogenesis is characterized by its ability to induce acute interstitial nephritis, tubular degeneration, and urate deposition, distinguishing it from strains that primarily infect the respiratory tract (8, 10). On the other hand, the Conn A5968 strain is known for its respiratory tropism, primarily affecting the trachea and upper respiratory tract (4, 11). This strain typically results in respiratory lesions such as tracheitis, bronchitis, and ciliostasis, without significant nephropathogenic effects (1). Understanding the specific tropism of these strains is crucial, as it highlights the diverse pathogenic mechanisms of IBV and underscores the necessity of tailored therapeutic strategies (12). Our study focuses on these distinct pathotypes to provide a clearer context for understanding IBV infections and emphasizes the relevance of our findings to both respiratory and nephropathogenic IBV strains.

Numerous studies have highlighted IBV’s capability to grow and replicate in tracheal organ explants (tracheal organ culture or TOC) without requiring prior adaptation (13). This *ex vivo* system, TOC, has proven to be a reliable method for isolating respiratory viruses, including IBV (9, 10). The demonstration of IBV replication in the respiratory tract of chickens has been confirmed in both *in vivo* tracheal tissues (14) and tracheal organ cultures (15). The primary effector of tracheal innate immunity against IBV involves goblet cell hyperplasia and alveolar mucous gland activation, resulting in seromucous nasal discharge and the formation of catarrhal or caseous exudates within the trachea (1). The interaction between respiratory viruses and chicken tracheal tissues triggers a cascade of immunomodulatory events, including leukocyte recruitment, release of pro-inflammatory cytokines, chemokines, and bioactive lipid mediators, particularly prostaglandins (PGs) (5, 16).

Comparatively, other avian respiratory infections, such as avian influenza virus (AIV) and Newcastle disease virus (NDV), exhibit both similarities and differences in their pathogenesis, immune responses, and clinical outcomes. For instance, AIV, like IBV, targets the respiratory epithelium but can also spread systemically, causing severe multi-organ disease in birds (17). AIV infection induces a robust immune response characterized by the production of type I interferons, pro-inflammatory cytokines, and the activation of both innate and adaptive immune pathways (18). NDV, another significant avian pathogen, primarily infects the respiratory and nervous systems, leading to respiratory distress, neurological signs, and high mortality rates in severe cases (19, 20). The immune response to NDV includes the activation of dendritic cells, natural killer cells, and the production of interferons and other cytokines, similar to the response seen with IBV (21). However, the clinical outcomes of these infections can differ markedly. IBV primarily causes respiratory signs and, in some strains, nephropathogenic disease, leading to kidney damage (22, 23). These differences underscore the unique aspects of IBV pathogenesis and highlight the importance of studying its replication and immune evasion strategies in tracheal organ explants (23). Understanding these mechanisms can provide insights into the development of targeted interventions and improve our broader knowledge of avian infectious diseases.

Prostaglandin (PG) E2 plays a central role in inflammation and immune regulation (24). Specifically, the induction of cyclooxygenase-2 (COX-2), a key enzyme involved in prostaglandin biosynthesis, has been implicated in the generation of PGE2 in response to IBV *in vivo* infection (25, 26). Previously, our study demonstrated that IBV infection in chicken macrophages significantly enhances the production of COX-2 and PGE2, along with increased expression of inducible nitric oxide synthase (iNOS), nitric oxide (NO), and interleukin-6 (IL-6). Inhibiting the COX-2/PGE2 pathway not only reduces IBV replication but also diminishes iNOS and IL-6 expressions (27), suggesting that targeting this pathway may serve as a potential antiviral strategy to control IBV infection in chickens.

Despite documented toll-like receptor (TLR) involvement in the immune response against AIV in tracheal explants (28), the mechanistic pathway governing IBV pathogenesis, particularly the downstream signaling leading to COX-2/PGE2 pathway activation in TOC, remains poorly understood. Therefore, this study aimed to unravel the localized engagement of the COX-2/PGE2 pathway in the pathogenesis of IBV infection in chicken trachea. A comprehensive understanding of the intricate interplay between IBV, tracheal inflammation, key pro-inflammatory cytokines such as interferon (IFN)-γ, Janus kinase (JAK)-signal transduction and activation of transcription (STAT), and the COX-2/PGE2 pathways is crucial for deciphering the mechanisms underlying IBV pathogenesis and developing targeted antiviral agents to mitigate the impact of IBV infections. Our research on IBV replication in TOCs thus offers a valuable perspective within the field of avian infectious diseases. By comparing the pathogenesis and immune responses elicited by different IBV strains, our study emphasizes the significance of elucidating the specific mechanisms of IBV infection. This knowledge is crucial for developing effective therapeutic strategies and enhancing the health and productivity of poultry.

## MATERIALS AND METHODS

### Reagents and media preparations

Dulbecco’s Modified Eagle Medium (DMEM; Millipore Sigma, St. Louis, Missouri, USA), RNA later Stabilization Solution (Thermo Fisher Scientific, Carlsbad, California, USA), and 10% formalin (VWR International, LLC, Radnor, Pennsylvania, USA) were utilized in this study. The TOC medium was composed of DMEM supplemented with L-glutamine, 1% penicillin/streptomycin, and 0.5% HEPES (Millipore Sigma, St. Louis, Missouri, USA). Additionally, 1X Dulbecco’s phosphate-buffered saline (DPBS; Gibco, Thermo Fisher Scientific, Waltham, Massachusetts, USA) was used.

All inhibitors, including the COX-2 inhibitor SC-236 (10 µg/mL), PGE2, PGE2 receptor EP2 antagonist TG4-155 (4 µM), EP4 receptor antagonist L1-161 (8 µM), as well as JAK-1 inhibitor 420099 (15 nM) and JAK-2 inhibitor SP600125 (40 nM), were procured from Millipore Sigma (St. Louis, Missouri, USA). Additionally, interferon-gamma (IFN-γ: 100 ng/mL) was purchased from Kingfisher Biotech, Inc. (St. Paul, Minnesota, USA). All concentrations were derived from our previous study (27).

### Preparation of chicken tracheal organ cultures (TOCs)

Preparation of TOCs to ensure that all experiments were conducted with standardized concentrations and that TOC batches were handled uniformly to minimize variability. To further ensure consistency, all reagents and solutions were prepared fresh for each experiment, and incubation times and conditions were strictly adhered to the laboratory experimental protocols.

The TOCs were derived from 20-day-old chicken embryos sourced from a specific pathogen-free flock (Canadian Food Inspection Agency, Ottawa, Ontario, Canada). Following euthanasia, the trachea was carefully isolated at the level of the larynx, dissected from surrounding tissues, and promptly transferred to a pre-warmed TOC medium (29, 30). Each trachea was secured at the larynx and flushed approximately 20 times with a warm culture medium, utilizing a blunt 18-gauge needle and a 5 mL syringe to remove any mucus. Subsequently, the trachea was positioned on sterile 55 mm diameter Whitman filter paper discs (Whitman, Camden, New Jersey, USA). Using a McIlwain tissue chopper (Mickle Laboratories Ltd, Surrey, UK), the trachea was then finely sliced into approximately 1 mm segments. The rings proximal to the larynx were excluded, and those chosen, demonstrating nearly uniform diameters, were subsequently seeded directly into 48-well culture plates with 0.5 mL of medium allocated per ring. The TOCs were incubated at 37°C in a humidified incubator (5% CO_2_) for 24 hours. Following incubation, ciliary movement was assessed by observing under an inverted microscope (Olympus Life Science, Shinjuku-ku, Tokyo, Japan) to confirm the viability of the tracheal rings.

### Infection of tracheal organ culture with IBV

Tracheal organ cultures (TOCs) were allowed to settle for 24 hours prior to any treatments. Following this period, TOCs were subjected to three thorough washes using 1× DPBS (Sigma-Aldrich Corporation, St. Louis, Missouri, USA) with pipetting for effective cleansing, and the TOC culture media was then refreshed. Subsequently, TOC was inoculated with IBV strains (DMV/1639 or Conn A5968) at a titer of 2.301 log_10_ EID50, as detailed previously (29, 30). After an hour of adsorption period, the inoculum was aspirated, and the tracheal rings were washed thrice with 1× DPBS. Subsequently, fresh culture media was added. Infected and non-infected tracheal rings (*n* = 24/group), along with culture supernatants, were collected at 3, 6, 12, 24, and 48 hours post-inoculation (hpi). At these specified time points, 0.5 mL of culture supernatant was collected from each well, with 250 µL stored in 1.7 mL tubes at −80°C for viral genome load quantification, and the remaining 250 µL placed in 0.5 mL tubes for further investigations. The tracheal rings were divided into two equal groups at each time point. Half of the rings (*n* = 12/group) were stored at −80°C in 1 mL RNA later for viral genome load quantification, whereas the other half (*n* = 12/group) was placed in 1 mL 10% formalin at room temperature.

### Modulating COX-2/PGE2 pathway during IBV infection in tracheal explants

All treatments were administered after 1 hour adsorption period. For the purpose of modulating the COX-2/PGE2 pathway during IBV infection in tracheal explants, the following were applied to the tracheal explants; exogenous PGE2 at a concentration of 5 µg/mL for 24 hours, positioned downstream of the COX-2 pathway, was introduced to assess its impact on the infection process. Simultaneously, the selective COX-2 antagonist SC-236 was administered at a concentration of 10 µg/mL for 24 hours to elucidate the specific role of COX-2 in this context. Exploring PGE2 receptors involved the use of type 2 and type 4 receptor inhibitors, and TG4-155 (4 µM) and L-161 (8 µM) were applied for 24 hours, respectively. Furthermore, tracheal explants were treated with recombinant chicken IFN-γ at a concentration of 100 ng/mL for 24 hours. This was combined with a 30 min pre-treatment with JAK inhibitors: 420099 (15 nM) for JAK-1 and SP600125 (40 nM) for JAK-2.

### Histopathology

The formalized tracheal rings were submitted to Diagnostic Services Unit (DSU) of the University of Calgary Faculty of Veterinary Medicine (UCVM) for making Hematoxylin and Eosin (H & E) sections and sections for immunohistochemistry. The sections were examined under light microscopy (Olympus BX51, Center Valley, PA, USA) to investigate any IBV-related lesions (Table S1). The lesions (degeneration of epithelial cells, loss of epithelial cells, deciliation, edema and/or congestion in lamina propria, and lymphocyte infiltration in lamina propria) were scored based on a previously conducted scoring system with some modifications (31). The lesions were classified as follows: no change (0), mild (1), moderate (2), or severe (3). The microscopic lesions used in the scoring system are explained in detail in Table S1.

### Immunohistochemistry

Immunohistochemical procedures were conducted in accordance with established protocols (32). Briefly, deparaffinization of sections attached to positively charged slides (VWR International, Radnor, PA, USA) was carried out in two changes of xylene, followed by rehydration through serial passages in descending concentrations of alcohol. To block endogenous peroxidase activity in tracheal rings, incubation with a 3% H_2_O_2_ solution (VWR International, Radnor, PA, USA) in absolute methanol (MilliporeSigma, Burlington, Massachusetts, USA) was performed for 10 minutes at room temperature. Viral epitopes were retrieved by subjecting the slides to heating in a 10 mM citrate buffer at pH 6.0 in a microwave at 850 W for 5 minutes. Non-specific binding was prevented using 2.5% goat serum (Sigma-Aldrich Corporation, St. Louis, Missouri, USA). The sections were then exposed overnight to mouse primary anti-IBV nucleoprotein (N) antibody (Novus Biological, Bio-Techne, Toronto, ON, Canada) diluted 1:500 in 2.5% goat serum within a humidified chamber at 4°C. Subsequently, the slides underwent two washes with Tris-buffered saline (TBS) for 5 minutes on a rocking platform. Tracheal rings were incubated with the secondary antibody, goat anti-mouse IgG (H + L) (Vector Laboratories Inc., Newark, CA, USA), for 30 minutes at room temperature. An ABC peroxidase kit and 3,3’-diaminobenzidine (DAB) substrate solution were employed following the manufacturer’s protocol (Vector Laboratories, Newark, CA, USA) for visualizing the antibody binding. After antibody detection, the slides were rinsed in distilled water for 3 minutes, counterstained by immersion in hematoxylin (Vector Laboratories, Newark, CA, USA) for 8 minutes, and blued by running distilled water for 30 minutes. Dehydration was achieved through immersion of the slides in an ascending series of alcohol, followed by two immersions in xylene (MilliporeSigma, Burlington, Massachusetts, USA), respectively. Finally, cover-slipping of the sections was performed using a mounting solution (Vector Laboratories, Newark, CA, USA).

IBV antigen-positive cells were detected by capturing images of the entire tracheal ring epithelium under 20× magnification lenses (Olympus BX51, Center Valley, PA, USA). Images were taken from five different fields of high intensity, and the percentages of positive cells were calculated based on the ratio of staining density to the number of hematoxylin-stained nuclei using ImageJ software (version 1.46 a, NIH, Bethesda, MD, USA), as described previously (32).

### RNA extraction, reverse transcription, and qPCR

Total RNA extraction from tracheal rings and culture supernatant fluid (SNF) was done using Trizol reagent and Trizol LS (Invitrogen Canada Inc., Burlington, ON, Canada), respectively, in accordance with the manufacturer’s recommendations. The reverse transcription process utilized the SuperScript IV Reverse Transcriptase Kit (Invitrogen Life Technologies, Carlsbad, CA, USA) with 1 µg of RNA, quantified using a nanodrop 1000 spectrophotometer (Thermo Scientific, Wilmington, DE, USA). Viral genome quantification was achieved through SYBR green-based quantitative reverse transcription-polymerase chain reaction (qRT-PCR), targeting the nucleocapsid (N) gene of IBV, as previously described (4, 33). The primer details of IFN-α, IFN-β, IL-1β, IL-6, and iNOS target genes and β-actin housekeeping gene are indicated in Table S2. The qPCR reaction mixture and protocol were detailed in accordance with established procedures, employing the DDCT method referred to using the comparative threshold cycle (Ct) method, often known as the ^ΔΔCt^ method. This technique is utilized to calculate relative expression levels of target genes compared with a reference or housekeeping gene, as previously documented (27, 28). Data were transformed to log10 copies of viral RNA using a standard curve generated from six 10-fold dilutions of an in-house prepared plasmid (27).

### Intratracheal COX-2 assay

During RNA extraction from tracheal rings, the organic phase underwent protein purification following the Trizol reagent according to the manufacturer’s protocol (Invitrogen Canada Inc., Burlington, ON, Canada). Protein concentration in the samples was determined using the Pierce BCA Protein Assay kit (Thermo Fisher Scientific, Waltham, Massachusetts, USA). Approximately 100 µg of purified protein from each tracheal ring was subjected to the COX-2 competitive Enzyme-linked Immunosorbent Assay (ELISA) assay using the ab210574 Mouse COX2 SimpleStep ELISA Kit (Abcam, Cambridge, Cambridgeshire, UK).

### Extratracheal PGE2 assay

The quantification of PGE2 levels in the culture SNF of in the culture supernatants of tracheal rings at the indicated time points was carried out using the PGE2 assay kit (R&D Systems, Minneapolis, Minnesota, USA). The assay was conducted in accordance with the manufacturer’s instructions, incorporating blank wells, standards, and both positive and negative controls. Absorbance readings were obtained at 450 nm using a horizontal microplate reader (680 XR, Microplate Reader, Bio-Rad, Hercules, California, USA), and PGE2 concentrations in the culture SNF were determined based on the constructed standard curve.

### Statistical analysis

The statistical analyses for this study were performed using GraphPad Prism software (version 9.5.1; 733, San Diego, California, USA). To evaluate the collective impact of the two distinct IBV strains at various time points, a two-way analysis of variance (ANOVA) was executed, and for the analysis of drug effects at 24 hpi, a one-way ANOVA was employed. Normality of the data residuals was assessed using the Shapiro-Wilk test in GraphPad Prism. The Bonferroni post hoc test was then applied for pairwise comparisons between groups. Significance was determined at *P* < 0.05.

## RESULTS

### Replication dynamics of IBV in tracheal rings and culture supernatants

**Fig 1:**
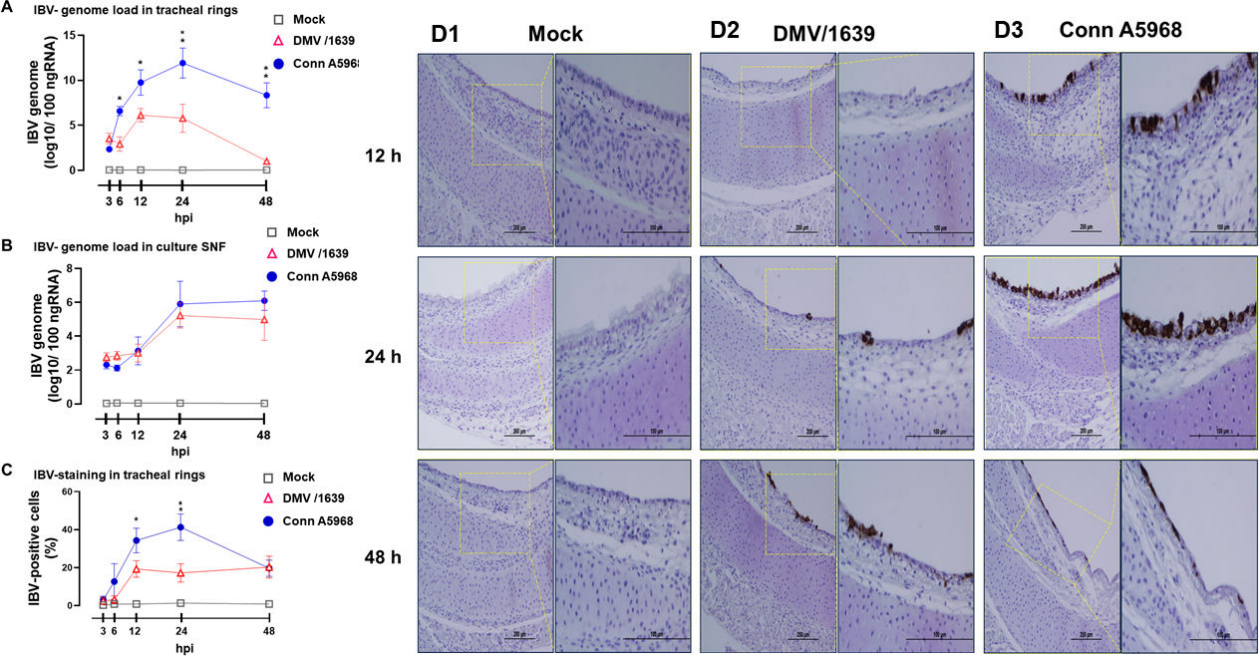
Quantification of infectious bronchitis virus (IBV) genome load and percentage of IBV-positive cells in tracheal explants. (**A**) IBV genome loads for two IBV strains, DMV/1639 and Conn A5968, were assessed at 3, 6, 12, 24, and 48 hours post-infection (hpi) using qPCR in tracheal rings. This analysis provided insights into viral replication within the tissue. (**B**) Simultaneously, IBV genome loads were quantified in culture SNF, offering information on viral shedding and release into the surrounding environment. (**C**) The percentage of IBV-positive cells in tracheal tissues was determined, providing valuable insights into the extent of viral replication within the tracheal mucosa. Data are presented as mean ± standard deviation from two independent experiments. Statistical significance was determined using a two-way ANOVA followed by Bonferroni post hoc tests (*P < 0.05, **P < 0.01, ***P < 0.001). Additionally, (**D**) immunohistochemistry images depict IBV-antigen staining at 12, 24, and 48 hpi for both IBV DMV/1639 (**D2**) and Conn A5968 strains (**D3**), in comparison to mock-inoculated controls (**D1**). The scale bar measures 200 µm for the left panel and 100 µm for the right panel.

Our initial experiments aimed to analyze the replication dynamics of IBV strains Conn A5968 and DMV/1639 in TOCs by quantifying viral genome loads and assessing IBV-positive cell percentages post-infection (Fig. 1). The IBV Conn A5968 viral genome load in tracheal rings was significantly higher compared with IBV DMV/1639 genome load at 6, 12, 24, and 48 hpi (Fig. 1A; *P* < 0.0001). The peaks of viral genome load were observed at 6 and 12 hpi for IBV DMV/1639 and IBV Conn A5968, respectively. However, the viral shedding, as denoted by culture SNF viral genome loads, was not significantly different between the two IBV strains at the indicated time points (Fig. 1B, *P* > 0.05). The percentage of IBV-positive cells within the tracheal rings was calculated based on the IBV-N antigen staining relative to nuclei at 3, 6, 12, 24, and 48 hpi (Fig. 1C). The immunohistochemical data confirmed the significantly higher expression pattern of the N-antigen for IBV Conn A5968 compared with IBV DMV/1639 at 12 and 24 hpi (*P* < 0.0001). The representative images of immunohistochemical staining of IBV antigens in tracheal rings are illustrated in Fig. 1D.

### Histological lesions induced by IBV in tracheal explants

**Fig 2:**
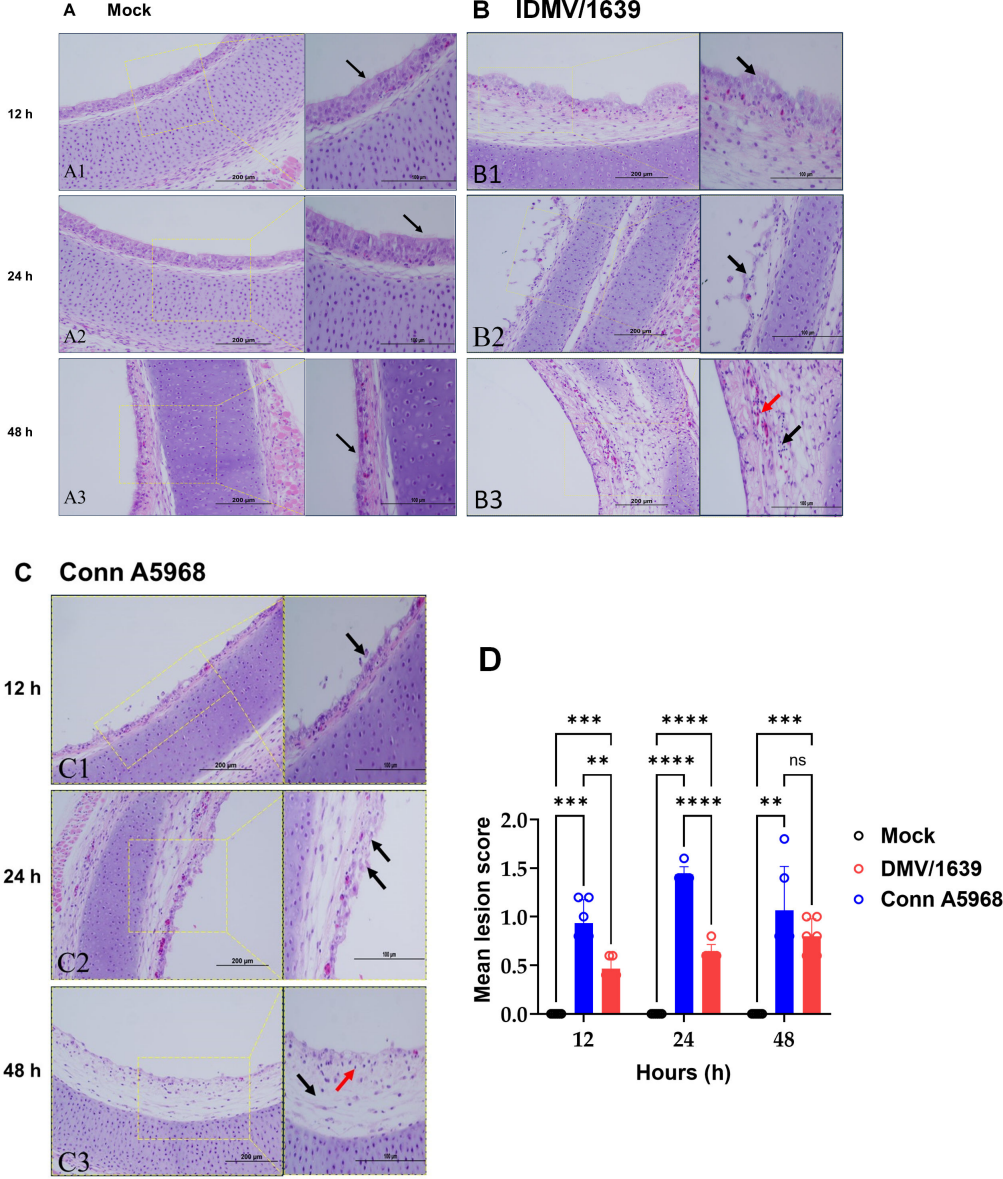
Histopathological lesions in tracheal explants induced by infectious bronchitis virus (IBV). Tracheal rings were harvested from tracheal organ culture (TOC) at 3, 6, 12, 24, and 48 hours post-infection (hpi) from IBV DMV/1639 or Conn A5968 inoculated groups and controls. (A1–A3) represent mock-infected control tracheal sections displaying normal histological architecture. (B1–B2) depict IBV DMV/1639-infected tracheal sections, with (B1) showing rounded-vacuolated epithelial cells and (B2) indicating sloughed epithelia and deciliation. (C1–C3) showcase IBV Conn A5968-infected tracheal sections, where (C1) demonstrates focal epithelial and ciliary losses, (C2) exhibits sloughed epithelia and deciliation in a wide area of mucosa, and (C3) reveals edema and lymphocyte infiltration marked by black and red arrows, respectively. The experiment was conducted twice and (D) represents the primary lesion scoring of 6 biological replicates. Analysis was performed using two-way ANOVA followed by Bonferroni post-test. Data represents means ± SD. Asterisks denote statistical significance at *P* < 0.05.

In the subsequent part of the results, the histopathological analysis revealed distinct temporal dynamics and lesion patterns in tracheal sections infected with IBV strains DMV/1639 and Conn A5968 (Fig. 2). There were no obvious microscopic changes in the uninoculated tracheal sections throughout the experiment (Fig. 2 Plates; A1-A3). However, histopathological findings of both IBV DMV/1639- and IBV Conn A5968-infected tracheal sections were clearly visible starting from 12 hpi to 48 hpi. At 12 hpi, some epithelia of the IBV DMV/1639-infected tracheal sections were rounded and exhibited vacuolar degeneration (Fig. 2 Plate; B1), whereas the epithelial lining of the IBV Conn A5968-infected tracheal sections displayed necrosis and deciliation with focal areas of epithelial sloughing (Fig. 2 Plate; C1).

By 24–48 hpi, the microscopic lesions became similar in the two IBV inoculated groups. However, the severity and extent of lesions varied between the groups. Both IBV DMV/1639- and IBV Conn A5968-infected tracheal sections revealed wide areas of epithelial and ciliary losses along the mucosal epithelium at 24 hpi (Fig. 2 Plates; B2, C2). By 48 hpi, lymphocytic infiltration and circulatory disturbances including edema and congested blood capillaries were evident in the lamina propria of both-IBV infected tracheal sections (Fig. 2 Plates B3, C3).

Regarding the histopathological scores, both IBV DMV/1639- and Conn A5968-infected tracheal sections had significantly higher lesion scores compared with the uninfected (mock) tracheal sections at 12, 24, and 48 hpi (Fig. 2D, *P* < 0.05). The lesion scores in the IBV Conn A5968-infected TOCs were significantly higher than those infected with IBV DMV/1639 at 12 and 24 hpi (*P* < 0.05). However, no significant differences could be detected between the two infected groups at 48 hpi (*P* > 0.05).

### Effect of modulating COX-2/PGE2 pathway in tracheal explants on IBV genome load

**Fig 3:**
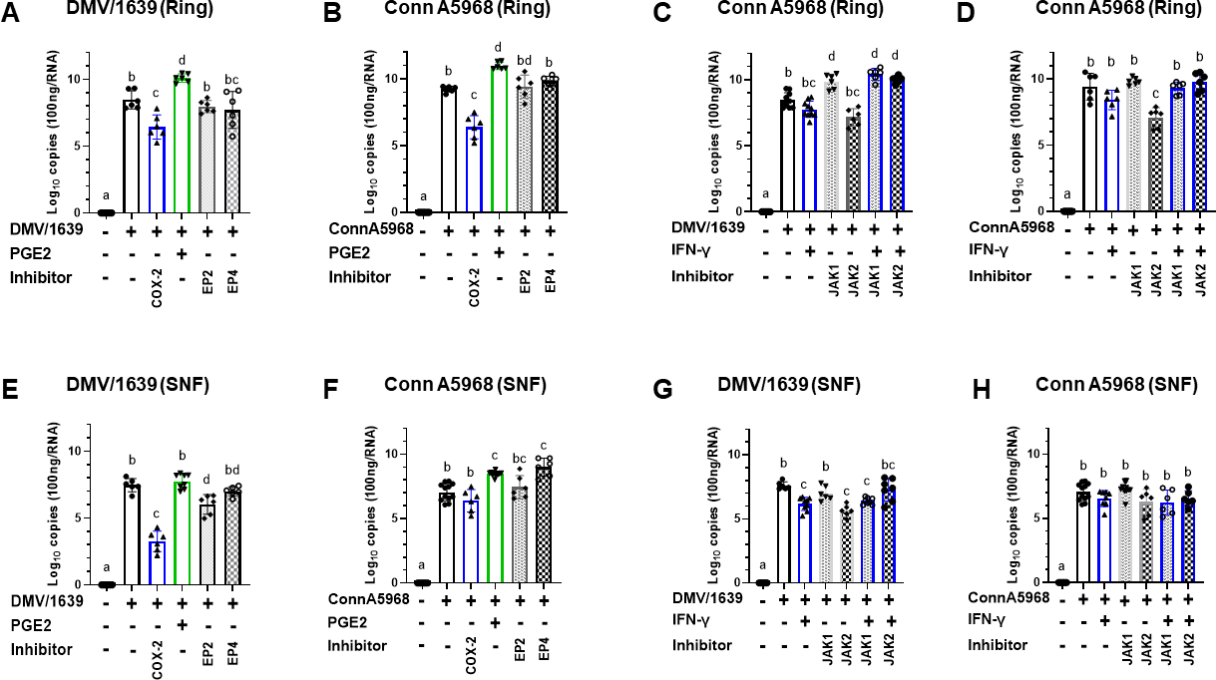
IBV genome loads in tracheal rings and culture supernatants (SNF) in response to various modulators. IBV genome loads were evaluated for two viral strains, DMV/1639 and Conn A5968, following a 1 h adsorption period and subsequent treatment with exogenous PGE2 (5 µg/mL), recombinant chicken IFN-γ (100 ng/mL), or inhibitors targeting COX-2 (SC-236; 10 µg/mL, EP2 (TG4-155; 4 nM), EP2 receptors (L1-61; 8 nM), JAK-1 (420099;15 nM), and JAK-2 (SP600125; 40 nM) in tracheal rings. (**A–D**) IBV genome loads were quantified at 24 h post-infection (hpi), providing insights into viral replication within the tracheal rings and the impact of specific inhibitors within the tissue. Similarly, IBV genome loads were quantified in SNF ((**E–H**), reflecting viral release into the culture medium under the same experimental conditions. Data are presented as mean ± standard deviation from one of two independent experiments. Values with different superscripts indicate significant differences (*P* < 0.05).

Then, we treated TOCs with inhibitors and modulators following IBV infection to investigate how COX-2/PGE2 pathway modulation affects IBV replication dynamics in tracheal rings and culture supernatants, aiming to clarify their regulatory roles in tracheal IBV infection (Fig. 3). With respect to the non-infected TOCs followed by incubating drugs for 24 h, all drug-treated TOCs groups revealed normal histological architecture in tracheal sections as previously described (Fig. 2 Plate A2). On the other hand, there were microscopic changes in the IBV-infected and drug-treated tracheal sections.

The treatment with COX-2 antagonist, SC-236 (10 µg/mL), was utilized to investigate COX-2′s role in IBV infection in TOCs (Fig. 3). At 24 hpi, a significant reduction in tracheal ring genome loads was observed following SC-236 treatment for both IBV Conn A5968 and IBV DMV/1639 strains (Fig. 3A and B*; P* < 0.0001). In the culture SNF, IBV genome loads significantly decreased after SC-236 treatment for the IBV DMV/1639 strain (Fig. 3E; *P* < 0.0001), but no significant change was observed for the IBV Conn A5968 strain (Fig. 3F; *P* > 0.05).

In contrast, a 24 h treatment with exogenous PGE2 resulted in a substantial increase in tracheal ring genome loads for both IBV DMV/1639 and IBV Conn A5968 strains compared with the non-treated control (Fig. 3A and B; *P* < 0.0001, *P* < 0.001, respectively). In the culture SNF, IBV genome loads were also elevated after 24 h PGE2 treatment for the IBV Conn A5968 strain (Fig. 3F; *P* < 0.0001), but no significant change was observed for the IBV DMV/1639 strain (Fig. 3E; *P* > 0.5).

Regarding the 24 h treatment with PGE2 receptor inhibitors, EP2 (TG4-155; 4 nM) and EP4 (L1-61; 8 nM), no reductions were noted in tracheal ring IBV genome loads for both IBV DMV1639 and IBV Conn A5968 strains (Fig. 3A and B, *P* > 0.05). However, a significant reduction was observed in IBV genome loads in culture SNF after treatment with EP2 inhibitor TG4-155 for the IBV DMV/1639 strain, and an increase after treatment with EP4 inhibitor L1-61 for the IBV Conn A5968 strain (Fig. 3E and F, *P <* 0.001, *P <* 0.001, respectively).

Considering the role of IFN-γ in enhancing antiviral responses, we aimed to investigate the combined effect of IFN-γ and JAK inhibition on IBV infection. We first examined the ability of IBV to induce IFN-γ *in vitro* and *ex vivo*, using a splenic-derived chicken macrophage cell line and TOCs, respectively. Both IBV DMV and IBV Conn A5968 strains significantly increased the concentration of IFN-γ in the culture SNF of macrophage cells at 3, 6, 12, and 24 hpi (Supplementary Material; Fig. S1A). However, this significant increase was not observed in the culture SNF of TOCs (Fig. S1A). In parallel, TOCs underwent a 24 h exposure to recombinant chicken IFN-γ (100 ng/mL), resulting in a noteworthy reduction in viral genome loads in tracheal rings and culture SNF for IBV strain, DMV/1639 (Fig. 3C and G; *P* = 0.003, *P* < 0.001, respectively) but not for the IBV strain, Conn A5968 (Fig. 3D and H; *P* > 0.05). Then, we employed inhibitors targeting the IFN-γ signaling pathway by treating IBV-infected TOCs with chemical antagonists to JAK-1 and JAK-2; 420099 (15 nM) and SP600125 (40 nM), respectively (Fig. 3C, D, G, and H).

A 24 h treatment of IBV DMV/1639 and IBV Conn A5968-infected TOCs with the JAK-2 inhibitor SP600125 significantly reduced IBV genome loads in tracheal rings (Fig. 3E and G, *P* < 0.0001, *P* < 0.001, respectively). However, in the culture SNF, IBV DMV1639 genome load was reduced (Fig. 3F, *P* = 0.0087) but not the IBV Conn A5968 genome load following JAK-2 inhibitor SP600125 treatment (Fig. 3H, *P* > 0.05). On the contrary, pronounced effects on IBV DMV1639 and Conn A5968 genome loads were not evident following a 24 h treatment with the JAK-1 inhibitor, 420099, either in IBV infected tracheal rings or culture SNF (Fig. 3E through H, *P* > 0.05). To further confirm the effects of IFN-γ, we treated IBV DMV1639 and IBV Conn A5968 infected TOCs with JAK-1 and JAK-2 inhibitors 30 min before the application of IFN-γ. The reduction in IBV DMV1639 genome load observed in tracheal rings and culture SNF following JAK-2 inhibitor treatment was reversed, bringing the viral loads to a level observed in IBV DMV1639 infected and non-treated controls (Fig. 3E and F, *P* = 0.025, *P* = 0.032, respectively). The reduction in IBV Conn A5968 genome load observed in tracheal rings following JAK-2 inhibitor treatment was reversed, bringing the viral loads to a level observed in IBV Conn A5968 infected and non-treated controls (Fig. 3G, *P* < 0.001).

### Effect of modulating COX-2/PGE2 pathway in tracheal explants on IBV replication and histopathology

**Fig 4:**
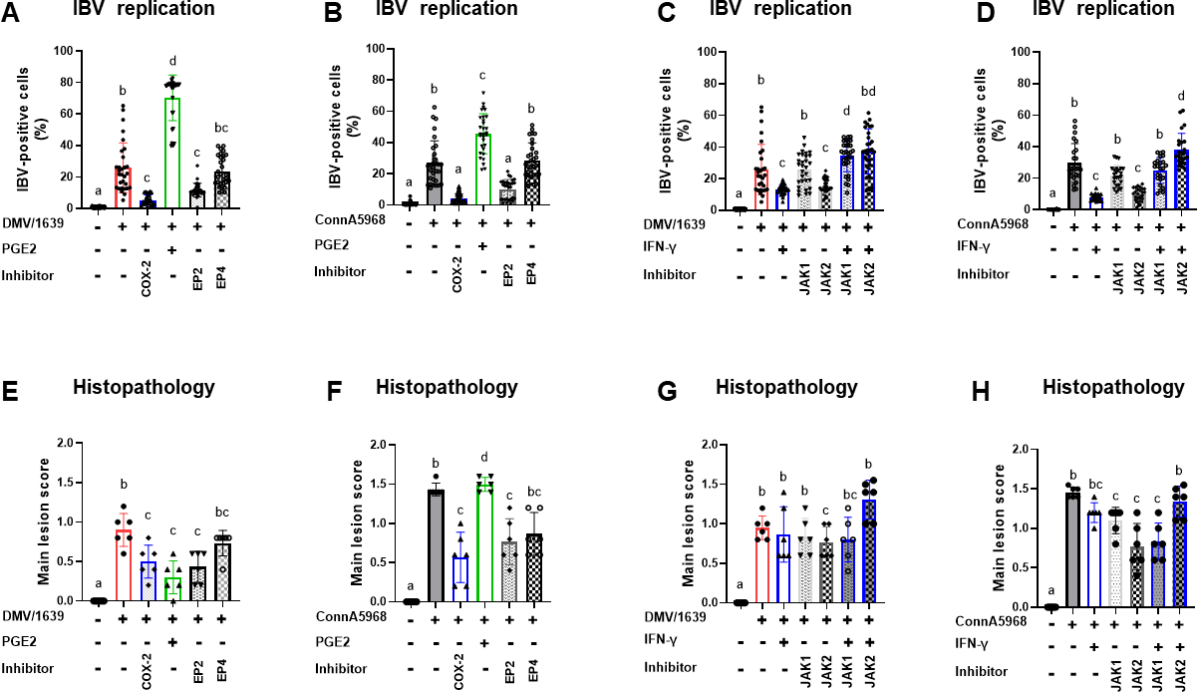
IBV replication and histopathological changes in tracheal explants in response to treatment with various modulators**.** Evaluation of IBV positive areas in the tracheal epithelium was conducted for two viral strains, DMV/1639 and Conn A5968, following a 1 h adsorption period and subsequent treatment with exogenous PGE2 (10 µg/mL), recombinant chicken IFN-γ (100 ng/mL), or inhibitors targeting COX-2 (SC-236; 10 µg/mL), EP2 (TG4-155; 4 nM), EP4 receptors (L1-61; 8 nM), JAK-1 (420099;15 nM), and JAK-2 (SP600125; 40 nM) in tracheal organ culture. (**A–D**) IBV-positive cells were calculated 24 h post-infection (hpi), providing insights into viral replication within the tracheal rings and the impact of specific inhibitors within the tissue. (**F–H**) Main lesion score under the same experimental conditions. Data are presented as mean ± standard deviation of two independent experiments. Values with different superscripts indicate significant differences (*P* < 0.05).

Examining the impact of modulating the COX-2/PGE2 pathway on IBV replication and histopathology in TOCs, our results in Fig. 4 provide insights into how targeted interventions alter viral replication dynamics and histological outcomes. Notably, a significant reduction in IBV-positive areas in the tracheal epithelium was observed after 24 h treatment with inhibitors targeting COX-2 and PGE2 pathways for both IBV strains (Fig. 4A and B, *P* < 0.0001). Conversely, exogenous treatment with PGE2 led to an increase in IBV-positive areas in the tracheal epithelium (Fig. 4A and B, *P <* 0.05). Additionally, a 24 h treatment with IFN-γ resulted in a reduction in the number of IBV-positive areas in the tracheal epithelium compared with the IBV-infected non-treated groups (Fig. 4C and D, *P <* 0.0001). However, this reduction was reversed when the 24 h treatment with IFN-γ was preceded by a 30-minute treatment with JAK-2 antagonists. (Fig. 4C and D, *P <* 0.05). Furthermore, treatment with JAK-2 alone exhibited a notable reduction in viral positive areas, whereas the effect was less apparent with JAK-1 treatment. These findings highlight the intricate modulation of IBV replication by targeted drug interventions, underscoring the potential therapeutic avenues for controlling IBV propagation.

The representative imaging of histopathology in tracheal rings in both IBV DMV/1639- and IBV Conn A5968-infected TOCs subjected to drug treatments is depicted in (Fig. S2A1 through 10 and Fig. S2B1 through 10). Treating IBV DMV/1639-infected TOCs with IFN-γ showed epithelial cell necrosis and epithelial cuffs inside the tracheal lumen (Fig. S2A6). Although the Conn A5968-infected TOCs then treated with IFN-γ were associated with intracellular vacuoles of epithelia; some nuclei of epithelial cells were fragmented or karyorrhectic (Fig. S2B6). In the IBV DMV/1639-infected TOCs with JAK-1 (420099) treatment, there were few epithelial cells with vacuolar degeneration Fig. S2A7); however, those infected with Conn A5968 revealed marked epithelial and ciliary losses (Fig. S1B7). Some epithelial cells suffered from vacuolation and deciliation in the IBV DMV/1639- and IBV Conn A5968-infected TOCs with JAK 2 treatment (SP600125) (Fig. S2A8). In addition, the lamina propria of the IBV Conn A5968-infected TOCs had some congested blood vessels (Fig. S2B8). The combination of the IFN-γ with either JAK-1 or JAK-2 in the DMV/1639-infected TOCs resulted in focal areas of epithelial and ciliary losses (Fig. S2A9 and A10). However, the latter combination was associated with morphological changes including lymphocyte infiltration, edema, and congestion in the lamina propria of the IBV Conn A5968-infected TOCs. Furthermore, focal epithelial and ciliary losses could be evident (Fig. S2B9 and 10).

In Fig. 4 (E through H), the IBV DMV/1639-infected TOCs then treated with COX-2 inhibitor, EP2, or JAK-2 inhibitors demonstrated significantly lower lesion scores than those infected only with DMV/1639 (Fig. 4E and G, *P* < 0.05). However, the IBV DMV/1639-infected TOCs followed by PGE2 or JAK- 2 + IFN-γ treatment revealed significantly higher lesion scores than those infected with DMV/1639 only (Fig. 4E and G, *P* < 0.05). Moreover, combining IFN-γ with either JAK-1 or JAK-2 in IBV DMV/1639-infected TOCs resulted in focal losses, whereas IBV Conn A5968-infected TOCs displayed morphological changes, including lymphocyte infiltration, edema, and congestion (Fig. S2A9 through B10). TOCs infected with IBV DMV/1639 and treated with all other treatments showed no significant differences from those only infected with IBV DMV/1639 (Fig. 4E and G, *P* > 0.05). TOCs treated with anti-COX-2, EP2, or JAK-2 in IBV DMV/1639-infected TOCs had significantly lower lesion scores than those infected only with DMV/1639 (Fig. 4E and G, *P* < 0.05). However, IBV DMV/1639-infected TOCs followed by PGE2 or JAK-2 + IFN-γ treatment had significantly higher lesion scores than those infected with IBV DMV/1639 only (Fig. 4E and G, *P* < 0.05). These results underscore the complexity of the antiviral response and highlight the need for careful consideration of treatment combinations to effectively manage IBV infections.

In IBV Conn A5968-infected TOCs, treatment with PGE2 did not result in significant differences in mean lesion scores compared with IBV Conn A5968-infected TOCs without treatment (Fig. 4G, *P* > 0.05). Similarly, no significant differences were observed between IBV Conn A5968-infected TOCs treated with IFN-γ + JAK-2 antagonists and those infected without treatment (Fig. 4H, *P* > 0.05). Conversely, treatment with inhibitors of COX-2, EP2, EP4, JAK-1, JAK-2, or JAK-1 combined with IFN-γ in IBV Conn A5968-infected TOCs resulted in significantly lower lesion scores compared with IBV Conn A5968-infected TOCs without treatment (Fig. 4G and H, *P* < 0.05). These findings suggest that targeted inhibition of specific pathways can effectively reduce lesion severity in IBV Conn A5968-infected TOCs.

Overall, our study highlights the differential responses of IBV strains to various treatments, emphasizing the importance of tailored therapeutic strategies.

### Effect of pharmacological inhibitors to COX-2 and JAK on IBV-mediated COX-2/PGE2 production in tracheal explants

**Fig 5:**
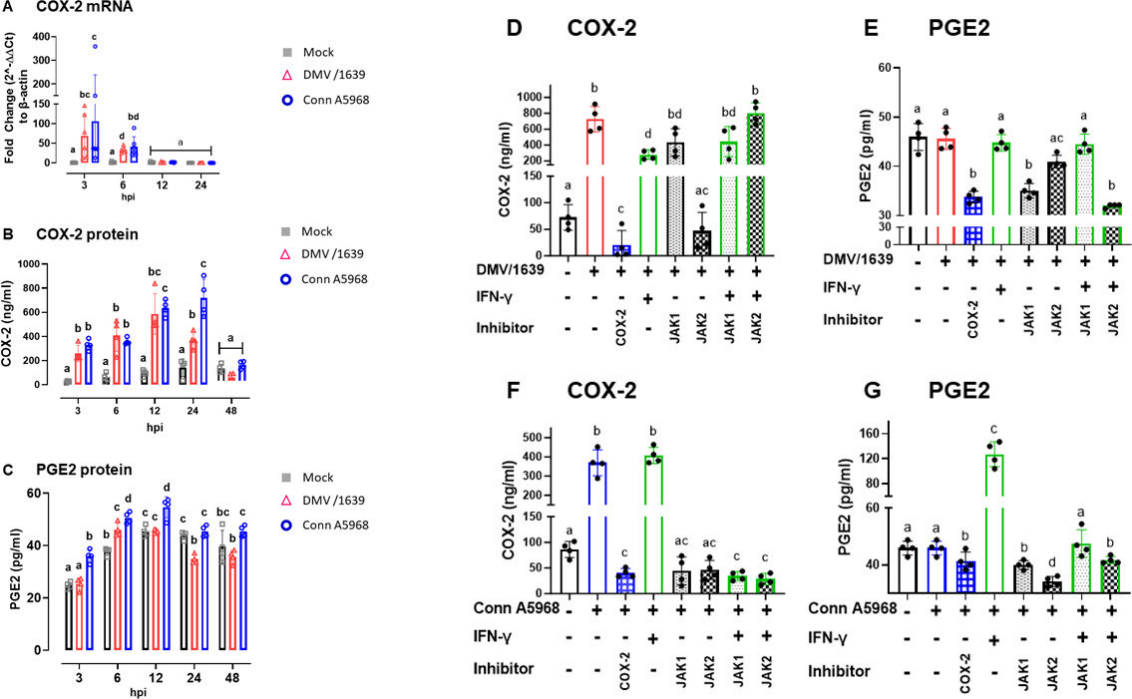
Modulation of COX-2 and PGE2 production in IBV-Infected tracheal rings and culture supernatants (SNF). Tracheal rings and culture SNF were harvested from tracheal organ cultures (TOC) at distinct time points (3, 6, 12, 24, and 48 hours post-infection, hpi) following IBV DMV/1639 or Conn A5968 inoculation, along with corresponding control groups. (**A–C**) Intratracheal COX-2 mRNA expression was quantified by qPCR, and COX-2 protein and extratracheal PGE2 levels were measured using ELISA kits. (**C–F**) Tracheal rings, subjected to a 1 h adsorption period, underwent treatment with exogenous PGE2 (5 µg/mL), recombinant chicken IFN-γ (100 ng/mL), or inhibitors targeting COX-2 (SC-236; 10 µg/mL), EP2 (TG4-155; 4 nM), EP4 receptors (L1-61; 8 nM), JAK-1 (420099;15 nM), and JAK-2 (SP600125; 40 nM). COX-2 protein levels (**C, E**) and PGE2 concentrations in culture SNF (**D, F**) at 24 hpi were quantified, offering insights into COX-2 synthesis within tracheal rings and its impact on PGE2 production. Data represent mean ± standard deviation of two independent experiments (A, *n* = 6), or of one of two independent experiments (B-F, *n* = 4). Significant differences are indicated by different superscripts (*P* < 0.05).

For investigating COX-2 and PGE2 modulation in IBV-infected TOCs, our study delves into critical aspects of host-virus interactions, shedding light on their dynamics and potential therapeutic implications. In Fig. 5, we explored the dynamic modulation of COX-2 and PGE2 production in IBV-infected TOCs, shedding light on crucial aspects of the host-virus interaction. Tracheal rings and culture SNF were meticulously sampled from TOCs at 3, 6, 12, 24, and 48 hpi following IBV DMV/1639 or Conn A5968 inoculation, alongside corresponding control groups.

The data depicted in Fig. 4A revealed an augmented expression of COX-2 mRNA at 3 and 6 hpi with IBV Conn A5968, contrasting with the peak expression observed at 3 h hpi with IBV DMV/1639. Additionally, quantification of intratracheal COX-2 and extratracheal PGE2 levels using ELISA demonstrated consistent temporal patterns (Fig. 5B and C). In IBV Conn A5968-inoculated TOCs, the COX-2 protein was significantly higher at 3, 6, 12, and 24 hpi (Fig. 5B, *P <* 0.05), and concomitantly, PGE2 levels in culture SNF persistently increased from 3 hpi to 48 hpi compared with non-infected controls (Fig. 5C, *P <* 0.05). Conversely, infection with IBV DMV/1639 significantly increased COX-2 protein expression at 3 hpi (Fig. 5B, *P <* 0.05), and at 6 hpi, the PGE2 concentrations in culture SNF also rose significantly (Fig. 5C, *P <* 0.05).

Our results indicated that the mock-infected, JAK-treated TOCs did not differ significantly from the mock-non-treated controls. Specifically, we observed no significant differences in cytokine expression between these groups (Fig. S3). This suggests that the JAK inhibition in the mock-infected samples did not induce off-target effects or alter baseline cellular responses, thereby validating the specificity and effectiveness of the JAK inhibitors used in our experiments. Furthermore, the lack of significant differences reinforces the reliability of using these inhibitors to specifically target JAK-1 and JAK-2 pathways in our IBV-infected TOCs.

Subsequent investigations involved TOCs treated with recombinant chicken IFN-γ and inhibitors targeting COX-2, JAK-1, and JAK-2 (Fig. 5D through G). The selective COX-2 inhibitor, SC-236, markedly reduced the elevated COX-2 protein levels in TOCs at 24 hpi with both IBV DMV/1639 and IBV Conn A5968 strains (Fig. 5D and E, *P* < 0.0001). This inhibitory effect was also reflected in lowering PGE2 concentrations in culture SNF of IBV DMV/1639 (Fig. 5F, *P <* 0.0001), but not for IBV Conn A5968 strain (Fig. 5G, *P* = 0.25). In parallel, inhibitors to JAK-1 and JAK-2 reduced the IBV-induced elevation in COX-2 protein in both DMV/1639 and Conn A5968 strains (Fig. 5F and G, *P* < 0.0001). Notably, the IFN-γ-augmented elevation in COX-2 protein (*P* < 0.0001) in IBV DMV/1639-infected TOCs was reversed by a 30-minute pre-treatment with the JAK-2 inhibitor but not with the JAK-1 inhibitor (Fig. 4D, *P* = 0.02). Similarly, the IBV Conn A5968-induced COX-2 protein and PGE2 concentrations were markedly reduced by inhibitors targeting JAK-1 and JAK-2 (Fig. 5F and G, *P <* 0.0001). In a consistent pattern, the IFN-γ-enhanced COX-2 protein and PGE2 releases in the IBV Conn A5968-infected TOCs were not reversed by either JAK-1 or JAK-2 inhibitors (Fig. 5F and G, *P* > 0.05). Taken together, the presented data are pivotal in unraveling the complex interplay of COX-2/PGE2 as host factors during IBV infection, with significant differences underscored by different superscripts (*P* < 0.05), offering valuable insights for potential therapeutic interventions.

### Proinflammatory cytokine expression in response to treatment with various modulators in IBV-infected tracheal explants

Next, we measured the proinflammatory cytokine expression and iNOS mRNA levels in IBV-infected TOCs to understand the interplay between the COX-2/PGE2 pathway and the host immune response. We initiated our investigation by assessing the impact of various modulators on the expression of proinflammatory cytokines and iNOS mRNA in non-infected tracheal rings. Fig S3 illustrates the effects of a 24 h treatment of TOCs with recombinant chicken IFN-γ (100 ng/mL), exogenous PGE2 Fig. S3A and B, or inhibitors targeting COX-2 (SC-236, Fig. S2C), EP2 (TG4-155), EP4 receptors (L1-61), JAK-1 (420099) in Fig. S3C and D, and JAK-2 (SP600125) in Fig. S2E and F.

Initially, treatment with IFN-γ significantly enhanced mRNA expressions of IFN-α, IFN-β, and iNOS compared with non-treated TOCs (Fig. S3A, *P* < 0.05). Exogenous PGE2 demonstrated a notable increase only in IL-1β mRNA expression (Fig. S2B, *P* < 0.05). Enhanced expression of IFN-α and IFN-β was also observed after 24 h treatment with selective inhibitors to COX-2 (SC-236; 10 µg/mL, Fig. S2C, *P* < 0.05) or EP2 (TG4-155; 4 nM, Fig. S3D, *P* < 0.05). Conversely, treatment with EP2 inhibitor (L1-61; 8 nM) for 24 h reduced the expression of IFN-α and IFN-β compared with non-infected and non-treated TOCs (Fig. S3E, *P* < 0.05). Fig. S2F demonstrates that the JAK-2 inhibitor (SP600125; 40 nM) decreased the expression of IFN-α and IFN-β, whereas JAK-1 inhibitors (420099; 15 nM) did not show any effects (Fig. S3E).

**Fig 6:**
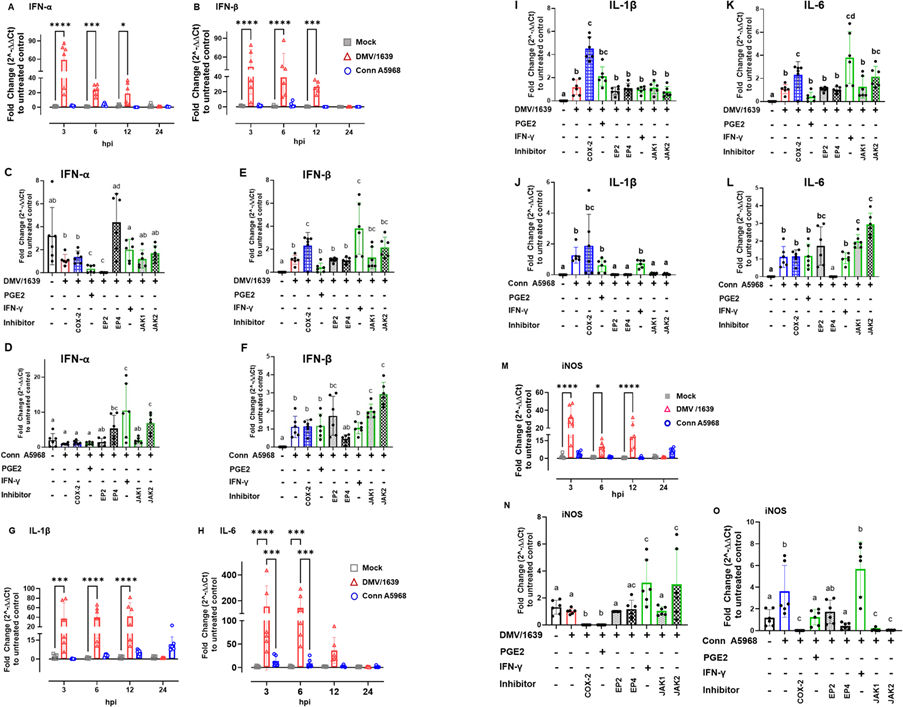
Modulation of IBV-Induced pro-inflammatory cytokine mRNA expression in chicken tracheal explants. RNA was extracted, and cDNA synthesis was performed to quantify relative mRNA expressions of IFN-α, IFN-β, IL-1β, IL-6, and iNOS through qPCR, using β-actin as the housekeeping gene. The analysis was conducted at indicated time points. Data from two independent experiments (*n* = 6) were subjected to two-way ANOVA, followed by Bonferroni post hoc test at 3, 6, 12, and 24 h post-infection (hpi) with IBV DMV/1639 and Conn A5968 strains (A, B, G, H, and M). Following 1 h of viral adsorption, rings were washed thrice, and TOC media were replaced with fresh media, followed by a 24 h incubation with the specified reagents: PGE2 (10 µg/mL), recombinant chicken IFN-γ (100 ng/mL), or inhibitors targeting COX-2 (SC-236; 10 µg/mL), EP2 (TG4-155; 4 nM), EP4 receptors (L1-61; 8 nM), JAK-1 (420099; 15 nM), and JAK-2 (SP600125; 40 nM) in tracheal organ culture (C-F, I-L, and O). Data in (C-F, I-L, and O) were analyzed by one-way ANOVA, followed by Bonferroni post hoc test, and are presented as mean ± standard deviation of two independent experiments (*n* = 6), all samples were run in duplicates. Values with asterisks or different superscripts indicate significant differences (*P* < 0.05).

Then, we investigated the effects of these modulators on cytokines mRNA expression in IBV-infected TOCs (Fig. 6). Infection of TOCs with IBV DMV/1639 led to a significant increase in IFN-α and IFN-β mRNA expressions at 3, 6, and 12 hpi (Fig. 6A and B, *P* < 0.05) compared with both IBV DMV/1639-infected TOCs and non-infected controls (Fig. 6A and B). The expression of IFN-α was reduced after 24 h treatment with PGE2 and inhibitors to EP2 (TG4-155) in IBV DMV/1639-infected TOCs (Fig. 6C, *P* < 0.05). In contrast, such expression was augmented by treatment with EP4 inhibitors (L-161), IFN-γ, and unaffected by inhibitors to JAK-1 and JAK-2, 420099, and SP600125, respectively. Similarly, IFN-α mRNA expressions were enhanced by the EP4 inhibitor and IFN-γ, also increased by JAK-2 (*P* < 0.05), but unaffected by other drugs (Fig. 6D). The IFN-β mRNA expressions induced by IBV DMV/1639 in TOCs (Fig. 6E) were further enhanced by 24 h treatment with the COX-2 inhibitor, SC-236 and IFN-γ, and remained unaffected by treatment with exogenous PGE2, as well as inhibitors to either EP2, EP4, or JAK-1 and JAK-2 (Fig. 6E, *P* < 0.05). However, the IFN-β mRNA expressions induced by IBV Conn A5968 were specifically enhanced by JAK-1 and JAK-2 inhibitors (Fig. 6F, *P* < 0.05).

In parallel, infection of TOCs with IBV DMV/1639 resulted in a marked increase in IL1-β mRNA expression at 3, 6, and 12 hpi (Fig. 6A, *P* < 0.05) and IL-6 mRNA expression at 3 and 6 hpi (Fig. 6B, *P* < 0.05) compared with both IBV Conn A5968-infected TOCs and non-infected controls (Fig. 6G and H). In IBV DMV/1639-infected TOCs, treatment with the COX-2 inhibitor resulted in an enhancement of IL1-β mRNA expression, whereas it remained unaffected by IFN-γ and other inhibitors, as depicted in Fig. 6I (*P* < 0.05). Similarly, in IBV Conn A5968-infected TOCs, the expression of IL1-β was notably increased by the COX-2 inhibitor, exhibiting no significant impact from PGE2 or IFN-γ treatments. Intriguingly, a significant reduction was observed with both EP and JAK antagonists, as illustrated in Fig. 6J (*P* < 0.05).

Concurrently, the infection of TOCs with IBV DMV/1639 resulted in a pronounced increase in IL1-β mRNA expression at 3, 6, and 12 hpi (Fig. 6A, *P* < 0.05) and IL-6 mRNA expression at 3 and 6 hpi (Fig. 6B, *P* < 0.05) compared with both IBV DMV/1639-infected TOCs and non-infected controls (Fig. 6A and B). In IBV DMV/1639-infected TOCs, the expression of IL1-β mRNA was reduced by exogenous treatment with PGE2 and EP2 inhibitor but enhanced by IFN-γ and the EP4 inhibitor (Fig. 6I, *P* < 0.05). In the same context, in IBV Conn A5968-infected TOCs, the expression of IL1-β was enhanced by IFN-γ and JAK-2 inhibitors as well (Fig. 6J, *P* < 0.05). On the other hand, the induced IL-6 mRNA expression in TOCs at 24 hpi with IBV DMV/1639 was significantly augmented by a 24 h treatment with COX-2 inhibitor and IFN-γ, whereas it remained unaffected by other treatments (Fig. 6K, *P* < 0.05). In the case of IBV Conn A5968-infected TOCs, the EP4 inhibitor reduced IL-6 mRNA expression, whereas JAK-1 and JAK-2 inhibitors enhanced such expressions (Fig. 6L, *P* < 0.05).

Infection of TOC with IBV DMV/1639 resulted in a significant increase in iNOS mRNA expression at 3, 6, and 12 hpi (*P* < 0.05). However, such a notable increase was not observed following infection with IBV Conn A5968 (Fig. 6M). A 24 h treatment with IFN-γ significantly augmented iNOS mRNA expression in TOCs infected with both IBV strains (Fig. 6N and O, *P* < 0.01). Interestingly, the JAK-2 inhibitor SP600125 increased iNOS mRNA expression in DMV-infected TOCs but not in the case of Conn A5968 strain, whereas the selective COX-2 inhibitor SC-236 reduced iNOS expression in both IBV strains (Fig. 6N and O).

Taken together, our results highlight the distinctive and dynamic patterns of IFN-α, IFN-β, IL1-β, IL-6, and iNOS mRNA expressions in response to IBV DMV/1639 infection, underscoring the intricate interplay between the virus and the host TOCs. The absence of a comparable response in IBV Conn A5968-infected TOCs further emphasizes the strain-specific distinctions in the host immune and inflammatory responses.

## DISCUSSION

This study delved into the distinct replication dynamics and innate immune responses of two distinct IBV strains, namely IBV Conn A5968 and IBV DMV/1639, within chicken TOCs. Our results revealed unique temporal profiles in viral genome loads, replication peaks, and IBV-antigen expression, emphasizing the individual pathogenic characteristics of each IBV strain in TOCs. Histopathological assessments provided insights into differential lesion scores, elucidating the comparative pathology induced by these IBV variants in TOCs. Notably, interventions targeting the COX-2/PGE2 pathway and JAK-2 demonstrated efficacy in mitigating lesion scores, suggesting promising antiviral avenues. Furthermore, exploration into the modulation of COX-2 and PGE2 production in IBV-infected TOCs offered critical insights into the host-virus interaction. Time-based analyses of COX-2 and PGE2 synthesis unveiled heightened COX-2 and PGE2 production, concomitant with elevated IBV Conn A5968 replication. This delineates the implication of this pathway in facilitating IBV replication. Experiments involving exogenous PGE2, recombinant chicken IFN-γ, and specific inhibitors on cytokine expression in TOCs revealed a diminished IBV DMV/1639 replication concurrent with elevated cytokine levels, particularly type-1 interferon and iNOS, providing an introductory understanding of host factors in IBV infection and potential antiviral strategies.

The replication dynamics of the virulent IBV M41-K and avirulent IBV Beau-R strains demonstrated a 24 hpi peak in culture SNF of chicken TOCs, followed by a subsequent decline (34). In contrast, our study revealed distinct replication peaks for IBV DMV/1639 and IBV Conn A5968 at 6 and 12 hpi, respectively, with no significant differences in extratracheal viral genome loads between the two strains. Immunolocalization of IBV antigen within tracheal rings’ epithelium and significantly higher lesion scores in IBV Conn A5968-infected TOCs at 12 and 24 hpi (Fig. 2) underscore the nuanced replication dynamics and distinctive features associated with each strain. These findings agree with Zhang et al. (15) and emphasize the multifaceted nature of viral dynamics and their potential impact on IBV pathogenesis. The observed discrepancy between the decrease in IBV load in tracheal rings and the stable load in the culture SNF may be due to the kinetics of viral replication, host cell turnover, and the effects of antiviral immune responses as reported in the respiratory tract of SARS-Cov patients (35).

These comprehensive histopathological examinations of IBV-infected TOCs offer valuable insights into the dynamic progression of infection and the impact of various drug interventions (36). Prior to drug interventions, the impact of selected dosages was assessed against a baseline of normal histological architecture. After ensuring that all TOCs groups subjected to 24 h treatments with selected drugs maintained an intact and standard histological structure in tracheal sections, in line with previously documented observations (Fig. S1). Antagonists targeting the COX-2 pathway, EP2, and JAK-2 demonstrate efficacy in reducing lesion scores (Fig. 3). On the other hand, drugs used in rescue experiments such as PGE2, and pretreatment of IFN-γ with JAK-2 inhibitor, exacerbated lesions and increased IBV replication (Fig. 3E through H), emphasizing the complexity of host-virus-drug interactions (37). These findings underscore the intricate nature of IBV infection and the potential of targeted interventions in modulating histopathological outcomes.

Then, we delved into the dynamic modulation of COX-2 and PGE2 production in IBV-infected TOCs, illuminating critical facets of the intricate host-virus interplay. Systematic sampling and data analysis at 3, 6, 12, 24, and 48 hpi with IBV DMV/1639 or Conn A5968, alongside respective control groups, provided a comprehensive temporal perspective for COX-2 and PGE2 synthesis within TOCs. Notably, our recent *in vitro* study revealed disparate induction patterns of COX-2/PGE2 in chicken macrophages, with the IBV DMV/1639 strain prevailing over IBV Conn A5968 (27). However, intriguingly, this TOCs model demonstrated an opposite effect (Fig. 4). Therefore, administration of exogenous PGE2, recombinant chicken IFN-γ, and inhibitors directed at COX-2, EP2 receptors, EP4 receptors, JAK1, and JAK2 was undertaken. Notably, a discernible decrease in COX-2 protein levels and PGE2 concentrations in culture SNF was observed at 24 h following treatment with selective antagonists targeting COX-2 and JAK-2 (Fig. 4C–F). These findings substantially enhance our comprehension of the precise molecular mechanisms governing COX-2 and PGE2 regulation in response to IBV infection, providing a foundation for potential antiviral agents used in managing IBV propagation.

The differential mechanisms of action between the JAK-1 inhibitor (420099) and the JAK-2 inhibitor (SP600125) are crucial for understanding their distinct impacts on IBV infection. Both inhibitors selectively block their respective targets within the JAK-STAT signaling pathway, which is essential for mediating immune responses against viral infections (38, 39), and JAK-1 antagonist (420099) primarily inhibits JAK-1 phosphorylation, disrupting the downstream signaling cascade initiated by cytokines like type I and II interferons, leading to a broad suppression of immune responses and a reduction in COX-2 and PGE2 levels in IBV-infected tracheal explants (40). However, 420099 may also affect other kinases and pathways, contributing to its differential impact on IBV infection (41, 42). In contrast, SP600125 targets JAK-2 and inhibits c-Jun N-terminal kinase (JNK), influencing stress and inflammatory responses (43). This dual inhibition affects cytokine and inflammatory mediator expression, potentially explaining the observed discrepancies in JAK-2 inhibition effects on IBV infection. Additionally, SP600125’s off-target effects on other kinases may uniquely impact viral replication and host immune regulation (43, 44). Understanding these distinct mechanisms and potential off-target effects is essential for elucidating how each inhibitor influences IBV immune regulation and underscores the complexity of the JAK-STAT pathway in antiviral responses (38). These insights are vital for developing targeted therapeutic strategies to modulate immune responses and control IBV infection in poultry.

The influence of the COX-2/PGE2 pathway on viral replication varies, depending on cell type and virus family. In line with this, PGE2 has been shown to promote IBV replication *in vitro* in macrophages (27) and enhance Marek’s disease virus replication both *in vivo* and *in vitro* (38, 45). Conversely, interventions targeting COX-2 and PGE2 production using selective inhibitors like NS-398 and celecoxib augmented NDV in chicken fibroblast cells (44). In our study, a 24 h treatment with inhibitors targeting COX-2 and the EP2 pathway mitigated viral replication in the TOCs (Fig. 5). This heightened release of IFN-γ aligns with existing literature that underscores the role of macrophages in mounting an early and potent immune response against viral infections including avian viruses like IBV (45, 46). Conversely, exogenous PGE2 administration increased viral propagation. Additionally, the antiviral effects of IFN-γ were observed with a 24 h treatment, and this effect was reversed by a 30 minute pre-treatment with JAK-2 antagonists, highlighting the role of the JAK-2 pathway in IFN-γ-mediated antiviral responses (Fig. 4 and 5). These findings underscore the intricate and divergent roles played by PGE2 in modulating viral infections across different cellular and viral contexts. Consistently, both IBV DMV/1639- and IBV Conn A5968-infected tracheal rings display marked mucosal desquamation and deciliation (Fig. S1), with interventions targeting COX-2, EP2, or JAK-2 demonstrating efficacy in reducing lesion scores in DMV/1639-infected TOCs (Fig. 5). In contrast, treatments with PGE2 or JAK-2 + IFN-γ resulted in significantly higher lesion scores in DMV/1639-infected TOCs compared with the infection-only group (Fig. 5). Conversely, treatments with PGE2 or JAK-2 + IFN-γ result in significantly higher lesion scores, highlighting the multifaceted interactions between PGE2, IFN-γ, and various inhibitors in modulating lesion severity induced by IBV infection.

Prior to examining the impact of immunomodulators on IBV infection in TOCs, our assessment commenced by investigating the individual effects of drugs on the TOCs system, in line with similar studies conducted on IBV infection in trachea and on TOCs infected with AIV (46). Our results revealed that IFN-γ treatment initiated a robust upregulation of IFN-α, IFN-β, and iNOS mRNA expressions, highlighting its crucial role in modulating key immune and pro-inflammatory pathways in the context of IBV infection (Fig. S2). Notably, exogenous PGE2 selectively upregulated IL-1β mRNA expression, suggesting a distinct regulatory mechanism. Furthermore, targeted interventions with COX-2 and EP2 inhibitors amplified the expressions of IFN-α and IFN-β, whereas EP4 inhibition demonstrated a suppressive effect (Fig. S2). Conversely, the JAK-1 inhibitor, 420099, showed a prominent increase in IFN-α and IFN-β expressions. Supporting this, treatment with recombinant chicken IFN-γ has been demonstrated to elevate the expression of proinflammatory cytokines such as IL-1-β, IL-6, and IL-8 in chicken monocytes, and induce the expression of IL-6, COX-2, and iNOS mRNA in IBV-infected chicken macrophage cell line (27). Furthermore, prior research has shown that 24 h after IBV infection, there is an increased expression of innate immune genes such as TLR3, TLR7, myeloid differentiation primary response 88 (MyD88), IL-1β, and IFN-β, as well as augmented recruitment of macrophages in the trachea and lungs (47, 48), and in chicken kidney cells (49). Noteworthy, substantial elevations in IL-1β and IL-6 mRNA expressions at earlier time points in IBV DMV/1639-infected TOCs highlight the distinct regulatory pathways governing cytokine responses, offering potential targets for therapeutic interventions.

Besides, the findings in Fig. 6 show that infection of TOCs with IBV DMV/1639 triggered a robust upregulation of IFN-α and IFN-β mRNA expressions at 3, 6, and 12 hpi, contrasting with the limited responses observed in both IBV Conn A5968-infected TOCs and non-infected controls. The intricate modulation of IFN-α and IFN-β was evident through distinct responses to pharmacological interventions, such as reduced IFN-α expression following treatment with PGE2 and EP2 inhibitors, augmented by EP4 inhibitors and IFN-γ, and unaffected by JAK-1 and JAK-2 inhibitors. Similarly, the induced IFN-β mRNA expressions in IBV DMV/1639-infected TOCs were further amplified by COX-2 inhibitor SC-236 and IFN-γ, remaining impervious to other treatments, whereas IBV Conn A5968-induced expressions of IFN-β mRNA were selectively enhanced by JAK-1 and JAK-2 inhibitors (Fig. 6F). In line with this finding, previous research has showcased the heightened expression of innate immune response-related genes such as IFN-α, IFN-β, IL-1-β, IL-6, iNOS, MYD88, TLR3, and TLR-7 in the chicken trachea following IBV infection (33, 50, 51). Additionally, investigations into the augmented innate immune response to IBV infection have been conducted both *in vivo* (33, 51) and *in vitro* using chicken models (10).

IBV DMV/1639 infection led to notable upregulation of IL-1β and IL-6 mRNA expressions at various early time points, diverging from IBV Conn A5968-infected TOCs and non-infected controls (Fig. 6). The regulation of IL-1β and IL-6 expressions showed distinct responses to drug interventions. COX-2 inhibitors have been demonstrated to suppress lipopolysaccharide-induced IL-1β expression in chicken macrophages *via* NF-κB and mitogen-activated protein kinase (MAPK) signaling pathway mediated by the protein p38 (p38 MAPK) pathway (15, 52). Conversely, treatment with PGE2 or COX-2 inhibitors increased IL-1β expression in IBV DMV/1639-infected TOCs, unaffected by IFN-γ. In contrast, IBV Conn A5968-induced IL-1β expression was potentiated by COX-2 antagonist and reduced by inhibitors to PGE2 receptors and JAK. Furthermore, IL-6 expression at 24 hpi with IBV DMV/1639 was significantly amplified by COX-2 inhibitor and IFN-γ, contrasting with distinct modulation in IBV Conn A5968-infected TOCs, where EP4 inhibitor reduced IL-6 expression, whereas JAK inhibitors enhanced it. These findings elucidate the regulatory pathways of IL-1β and IL-6 responses during IBV infection, suggesting potential therapeutic targets.

Furthermore, IFN-γ treatment markedly augmented iNOS mRNA expression in both IBV strains, revealing distinct effects of JAK-1 inhibitor in IBV DMV/1639 and selective inhibition by JAK-1 and JAK-2 antagonists in IBV Conn A5968-infected TOCs. These findings underscore the complex regulation of iNOS expression, offering potential targets for modulating host responses to IBV infection (Fig. 6N and O). Additionally, in line with prior research (16, 53), these results suggest that PGE2 modulates the host immune system by inducing immunosuppression, inhibiting nitric oxide (NO) production, and suppressing type-1 IFN expression, thereby enhancing IBV replication.

IBV typically induces respiratory illness, with the severity of the disease being modulated by both the chicken genotype and the specific IBV strain (52, 54). The respiratory strain represented by IBV Conn A5968 is distinguished by certain characteristics (4), whereas nephropathogenic strains like IBV DMV/1639, exemplified by the Australian T strain, have been associated with elevated mortality rates attributed to kidney failure, characterized by features such as mononuclear cell infiltration and inflammation (8, 53) or immunosuppression (54). In the context of IBV-infected tracheal organ explants, our findings highlight distinct characteristics between the divergent IBV strains DMV/1639 and Conn A5968. First, IBV DMV/1639 infection exhibited a robust induction of innate immune responses, evidenced by heightened expressions of IFN-α, IFN-β, IL-1β, IL-6, and iNOS mRNA. In contrast, IBV Conn A5968 infection was characterized by prominent COX-2 mRNA and protein expressions, accompanied by sustained PGE2 release. Despite IBV Conn A5968 showing comparatively higher replication than IBV DMV/1639 in TOCs, viral shedding into culture SNF was equivalent. Overall, these observations suggest that the IBV respiratory strain Conn A5968 may successfully evade the immune system, as evidenced by lower pro-inflammatory cytokine markers. Furthermore, its replication might be facilitated by an activated COX-2/PGE2 pathway. Conversely, heightened immune defense mechanisms against DMV/1639 strains appeared to restrict its replication, contributing to its clearance from tracheal explants.

Immunohistochemistry revealed that IBV replication, as indicated by IBV protein immunostaining, viral protein detection, and pathology, was evident at 12 hpi, peaked at 24 hpi, and subsequently declined (15). This decline at 48 hpi likely reflects regeneration from the germinal epithelium associated with viral shedding (53). Consequently, we believe this model is valid within the experimental parameters at 24 hpi, although its limitations may stem from non-respiratory strains of IBV and extended time points. This study enhances the understanding of the TOCs relevance and limitations, considering the immunopathological effects observed in our histopathological analysis and their influence on viral replication dynamics.

### Conclusions

Our study highlights differences between the IBV respiratory strain Conn A5968 and the nephropathogenic strain DMV/1639 in TOCs. IBV Conn A5968 showed higher replication with potential immune evasion *via* a less active proinflammatory response, likely involving the COX-2/PGE2 pathway. In contrast, IBV DMV/1639 induced stronger innate immune responses, limiting replication and promoting clearance. The COX-2 inhibitor SC-236 reduced viral genome loads and COX-2 protein levels, especially in DMV/1639-infected TOCs. PGE2 treatment increased viral loads, indicating its role in enhancing replication. EP2 and EP4 receptor inhibitors had varied effects, reflecting the complex PGE2 signaling. IFN-γ treatment significantly reduced viral loads and altered cytokine expression, with JAK-2 inhibition reversing these effects, whereas JAK-1 inhibition had minimal impact. Histopathological analysis confirmed these findings, showing that targeting the COX-2/PGE2 pathway reduced epithelial damage, whereas PGE2 treatment worsened it.

In summary, the COX-2/PGE2 pathway is a key modulator of IBV replication and host responses. COX-2 inhibitors and IFN-γ appear promising as antiviral agents, suggesting targeted therapies for IBV infections. Further *in vivo* studies are needed to explore these therapeutic implications.

## SUPPLEMENTAL MATERIAL

Supplemental material spectrum.00407-24-s0001.pdf Tables S1 and S2; Fig. S1 to S4.

ASM does not own the copyrights to Supplemental Material that may be linked to, or accessed through, an article. The authors have granted ASM a non-exclusive, world-wide license to publish the Supplemental Material files. Please contact the corresponding author directly for reuse.
