# Supplementary material for: Cyclooxygenase-2/prostaglandin E2 pathway orchestrates the replication of infectious bronchitis virus in chicken tracheal explants
Source: Microbiol Spectr. 2024 Oct 29;12(12):e00407-24. doi: 10.1128/spectrum.00407-24 (PMC11619240; doi:10.1128/spectrum.00407-24)
Supplement: Supplemental material — Tables S1 and S2; Fig. S1 to S4. [file spectrum.00407-24-s0001.pdf]

Supplemental Material

Innate Immune Responses and the Cyclooxygenase-2/Prostaglandin E2 Pathway Orchestrate the Replication of Infectious Bronchitis Virus in Chicken Tracheal Explants

Motamed Elsayed Mahmoud[a,b], Ahmed Ali [a,c], Muhammad Farooq[a], Ishara M. Isham [a], Sufna M. Suhail [a], Heshanthi Herath-Mudiyanselage [a], Ryan Rahimi [a], Mohamed Faizal Abdul-Careem a,*

[a] Faculty of Veterinary Medicine, University of Calgary, 3330 Hospital Drive NW, Calgary, AB, T2N 4N1, Canada

[b] Department of Animal Husbandry, Faculty of Veterinary Medicine, Sohag University, Sohag 84524, Egypt.

[c] Department of Pathology, Faculty of Veterinary Medicine, Beni-Suef University, Beni Suef, 62511, Egypt.

* Correspondence: Dr. Mohamed Faizal Abdul-Careem, Email: faizal.abdulcareem@ucalgary.ca; Phone +1-403-220-4462

Methodology

H &E sections from TOCs were examined under light microscopy (Olympus BX51, Center Valley, PA, USA) to investigate any IBV-related lesions (Table S1). The lesions were scored based on previously conducted scoring system with some modifications [1]. The lesions were classified as follow: no change (0), mild (1), moderate (2), or severe (3).

Table S1. The microscopic lesions used in the scoring system

Tissue	Microscopic lesions severity (0-3)
TOCs	Degeneration of epithelial cells<br>Loss of epithelial cells<br>Deciliation<br>Edema and/or congestion in lamina propria<br>Lymphocytic infiltration in lamina propria

Table S2. List of primers for target and housekeeping genes

Gene	Sequence (5′-3′)	Reference
**IBV**	F- GACGGAGGACCTGATGGTAA<br>R- CCCTTCTTCTGCTGATCCTG	[2]
**COX-2**	F- AGGACGGGCTATTATGGGGA<br>R-GTGATCTCGACGTCAACACG	[3]
**IFN-α,**	F- ATCCTGCTGCTCACGCTCCTTCTR<br>R- GGTGTTGCTGGTGTCCAGGATG	[4]
**IFN-β**	F- AGCAAGGACAAGAAGCAAGC<br>R- CGTGCCTTGGTTTACGAAGC	[4]
**IL-1β**	F-GTGAGGCTCAACATTGCGCTGTA<br>R-TGTCCAGGCGGTAGAAGATGAAG	[4]
**IL-6**	F-F-CAGGACGAGATGTGCAAGAA<br>R-TAGCACAGAGACTCGACGTT	[4]
**iNOS**	F- GGCAGCAGCGTCTCTATGACTTG<br>R-GACTTTAGGCTGCCCAGGTTG	[4]
**β-actin**	F- CAACACAGTGCTGTCTGGTGGTA<br>R- ATCGTACTCCTGCTTGCTGATCC	[4]

Interferon gamma Assay

Splenic-derived macrophage cell line (MQ-NCSU) cells were cultured and infected as previously described (5). The culture supernatant from both macrophages and tracheal explants was subjected to a competitive ELISA assay to measure the amount of Interferon gamma (IFN-γ) following IBV infection at 3, 6, 12, 24, and 48 hours post-infection (hpi), according to the manufacturer's instructions (Chicken IFN-gamma ELISA Kit, Invitrogen, Thermo Fisher Scientific, Carlsbad, CA, USA).

Statistical analysis

The relative expression data in supplementary Figure 2 were analysed by paired Student's t-test for group comparisons, values with asterisks mean $p < 0.05$.

Results and Discussion

Effect of IBV on Interferon gamma release

Compared to the enhanced release of IFN-γ following IBV infection at 3, 6, 12, and 24 hpi in splenic-derived macrophage cell lines (A), no significant increase in IFN-γ release was observed in tracheal organ cultures (TOCs) at any of the same time points (B). The macrophages showed a marked elevation in IFN-γ levels post-infection, indicating a robust immune response to IBV. In contrast, the TOCs did not exhibit a similar response, suggesting a tissue-specific variation in IFN-γ release or a different immunological interaction with IBV in tracheal tissues.

The observed data indicates a clear discrepancy in the immune response between splenic-derived macrophage cell lines and tracheal organ cultures (TOCs) following IBV infection.

Specifically, the splenic-derived macrophages demonstrated a significant increase in the release of Interferon gamma (IFN-γ) at 3, 6, 12, and 24 hours post-infection (hpi), highlighting a robust activation of the immune response. This heightened release of IFN-γ aligns with existing literature that underscores the role of macrophages in mounting an early and potent immune response against viral infections, including avian viruses like IBV (6-8).

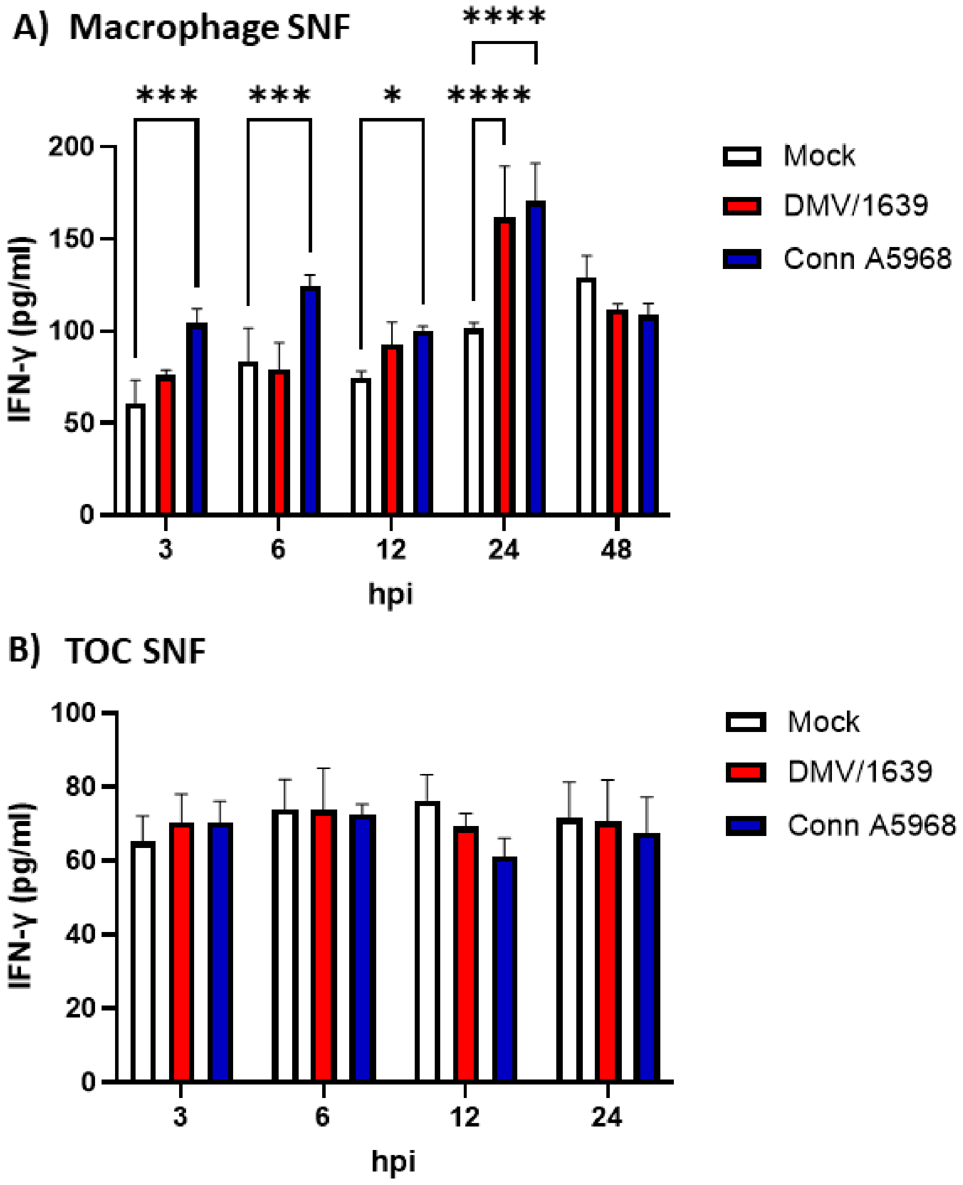

FIG S1. Interferon gamma release in culture supernatant following IBV infection of chicken macrophages and tracheal explants. The amounts of Interferon gamma (IFN-γ) were

quantified by competitive ELISA in culture supernatant of splenic-derived macrophage cell line (A) at 3, 6, 12, 24, and 48 hours post infection (hpi) and in tracheal organ culture supernatant at 3, 6, 12, and 24 hpi (B) with either DMV/1639 or Conn A5968 versus mock infected controls. The data represent mean ± SD. Analyses were performed by two-way ANOVA followed by Bonferroni **post**-test. $p < 0.05$ was considered significant.

In contrast, TOCs did not show a significant increase in IFN-γ release at any of the same time points. This lack of response in TOCs suggests a tissue-specific variation in immune response to IBV infection. The tracheal tissue, while still an integral part of the respiratory immune defense, may have a different cytokine release profile compared to macrophages. It is possible that other cytokines or immune mechanisms are more dominant in the tracheal tissue's response to IBV, as suggested by previous studies on respiratory mucosal immunity (9).

Additionally, the differences in immune response between these two cell types could be attributed to the local microenvironment and cellular interactions within the tissue. Macrophages, being part of the systemic immune system, may respond more vigorously to IBV due to their role in antigen presentation and cytokine production (10). In contrast, the epithelial cells and resident immune cells in the trachea might exhibit a more regulated or different immune response, possibly to avoid excessive inflammation that could damage the respiratory tissues (11). Further studies are needed to elucidate the exact mechanisms underlying this tissue-specific immune response to IBV. Investigating the role of other cytokines, signaling pathways, and cellular interactions in tracheal tissues could provide deeper insights into the localized immune defense against IBV.

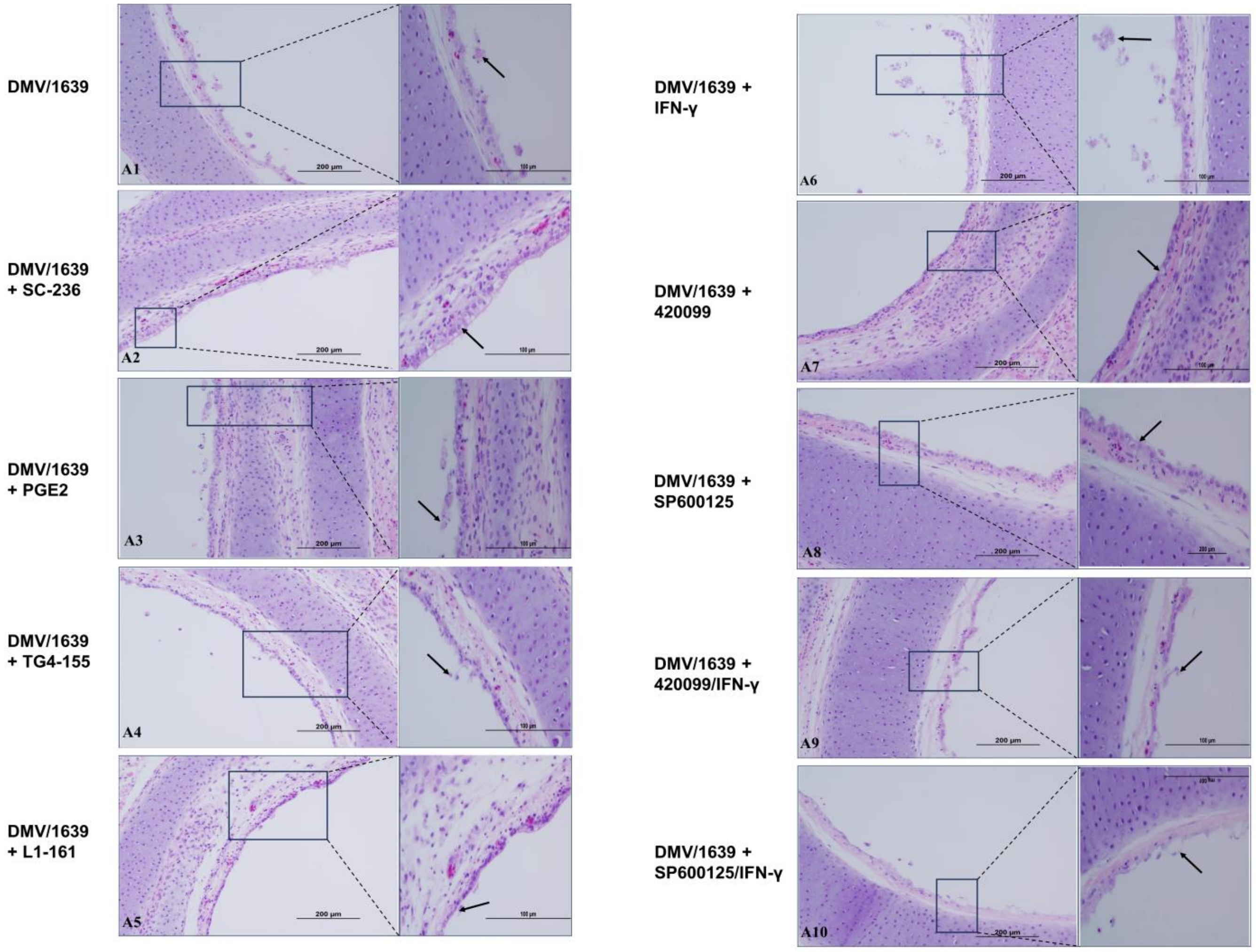

DMV/1639
DMV/1639 + SC-236
DMV/1639 + PGE2
DMV/1639 + TG4-155
DMV/1639 + L1-161
DMV/1639 + IFN-γ
DMV/1639 + 420099
DMV/1639 + SP600125
DMV/1639 + 420099/IFN-γ
DMV/1639 + SP600125/IFN-γ
A1
A2
A3
A4
A5
A6
A7
A8
A9
A10
200 μm
100 μm

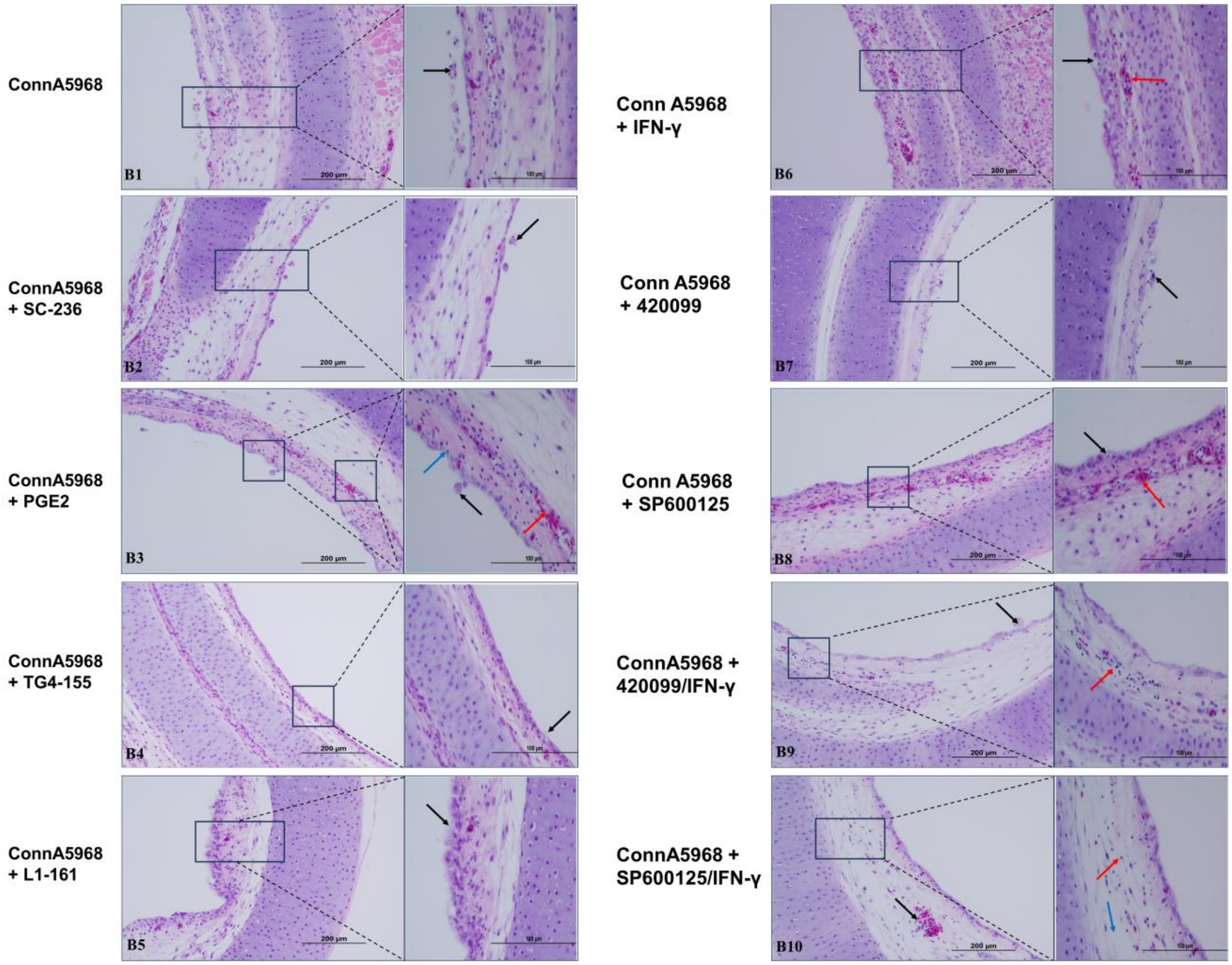

FIG S2 Representative Images of Histopathological Lesions in Tracheal Explants Treated with Modulators and Infected with IBV. Histological lesions were observed in tracheal rings harvested at 24 hours from tracheal organ cultures (TOCs) following infection with either DMV/1639 (A1-A10) or Conn A5968 (B1-B10) IBV strains and subsequent treatment with specific drugs. (A1): Black arrow denotes desquamated epithelia and deciliation; (A2): Black arrow highlights vacuolated epithelium; (A3): Black arrow indicates epithelial and ciliary losses; (A4 and A5): Black arrows depict a few sloughed-deciliated epithelia; (A6): Black arrow shows intra-luminal epithelial cell cuffs; (D7 and D8): Black arrows reveal vacuolated epithelial cells; (A9 and A10): Black arrows refer to focal epithelial and ciliary losses. (B1): Black arrow reveals extensive areas of epithelial desquamation and deciliation; (B2): Black arrow indicates focal epithelial and ciliary losses; (B3): Black arrow demonstrates sloughed epithelia, blue arrow shows intraepithelial lymphocytic infiltration, and red arrow reveals congested blood capillary; (B4): Black arrow indicates a few epithelia with deciliation; (B5): Black arrow shows vacuolated epithelial cells; (B6): Black arrow refers to vacuolated epithelia with fragmented and karyorrhectic nuclei, red arrow shows congested blood vessel; (B7): Black arrow refers to sloughed epithelia with ciliary loss; (B8): Black arrow indicates

vacuolar degeneration of some epithelia, red arrow reveals congested blood vessels; (B9): Black arrow shows focal sloughed epithelia, red arrow indicates lymphocytic infiltration; (B10): Red arrow reveals lymphocytic infiltration, blue arrow indicates edema.

Histopathological Findings

The uninfected tracheal organ cultures (TOCs) exhibited no evidence of microscopic changes at different time points (Fig. 2A1-A3) throughout the experiment. In contrast, both DMV/1639- and Conn A5968-infected TOCs displayed observable histopathological alterations from 12 to 48 hours post-infection (hpi). At 12 hpi, DMV/1639-infected TOCs exhibited rounded epithelia with vacuolar degeneration, while Conn A5968-infected TOCs showed necrosis and focal deciliation (Fig. 2B1, C1). From 24 to 48 hpi, both IBV-infected groups displayed widespread epithelial and ciliary losses (Fig. 2B2, C2). By 48 hpi, lymphocytic infiltration, edema, and congested blood capillaries were evident in the lamina propria of both infected TOCs (Fig. 2B3, C3).

Histopathological scores revealed significantly higher lesion scores in both DMV/1639- and Conn A5968-infected TOCs compared to uninfected TOCs at 12, 24, and 48 hpi ($P < 0.05$). Lesion scores were significantly higher in the Conn A5968-infected TOCs than the DMV/1639-infected TOCs at 12 and 24 hpi ($p < 0.05$), with no significant differences at 48 hpi ($p > 0.05$).

Upon incubating drugs for 24 hours in mock- or infected TOCs, all drug-treated TOCs with mock infection displayed normal histological architecture (Figure S1A2). Conversely, microscopic changes were observed in the IBV-infected and drug-treated TOCs.

At 24 hpi, the surface epithelium of DMV/1639- and Conn A5968-infected TOCs exhibited marked desquamation and deciliation (Figure S1; D1, E1). Anti-COX treatment in DMV/1639-infected TOCs showed intracytoplasmic vacuolation, while Conn A5968-infected TOCs with anti-COX treatment displayed sloughed epithelia with ciliary loss (Figure S1; D2, E2). PGE2 treatment in DMV/1639-infected TOCs resulted in necro-desquamation and deciliation, whereas Conn A5968-infected TOCs with PGE2 treatment exhibited epithelial loss, intraepithelial lymphocytic infiltration, lymphocytic infiltration, and congested blood vessels (Figure S1; D3, E3). TOCs treated with TG4-155 (EP2) exhibited few epithelial and ciliary losses, while TOCs treated with L1-161 (EP4) showed few epithelial cells with deciliation or epithelial cell vacuolation in DMV/1639- or Conn A5968-infected TOCs, respectively (Figure S1; D4-E5).

Treatment with interferon-gamma resulted in epithelial cell necrosis and cuffs in DMV/1639-infected TOCs, while Conn A5968-infected TOCs showed intracellular vacuoles, fragmented nuclei, and karyorrhectic changes (Figure S1; D6, E6). JAK1 treatment in DMV/1639-infected TOCs exhibited few epithelial cells with vacuolar degeneration, whereas Conn A5968-infected TOCs displayed marked epithelial and ciliary losses (Figure S1; D7, E7). JAK2 treatment in both infected TOCs showed epithelial and ciliary losses, with Conn A5968-infected TOCs displaying congested blood vessels (Figure S1; D8, E8).

The surface epithelium of the DMV/1639- and Conn A5968-infected tracheal rings was characterized by focal areas of marked desquamation and deciliation of the mucosa (Figure S1 A, C E, G, and Figure S1D1, E1). In the DMV/1639-infected TOCs followed by anti-COX inhibitor incubation, the epithelium showed intracytoplasmic vacuolation (Figure S1C, D2),

whereas the Conn A5968-infected TOCs with presence of COX-2 inhibitor had few sloughed epithelia with ciliary loss (Figure S1A, Figure S1E2). The DMV/1639-infected-TOCs were then treated with PGE2 revealed marked necro-desquamation and deciliation in multi-focal areas of the mucosa (Figure S1 C, Figure S1D3). While the Conn A5968-infected TOCs followed by PGE2 incubation were identified by few epithelial loss and intraepithelial lymphocytic infiltration. In addition, there were lymphocytic infiltration and congested blood vessels in the lamina propria (Figure 5E3). In both DMV/1639- and Conn A5968-infected TOCs then treated with TG4-155 (EP2), the lining epithelium had few epithelial and ciliary losses (Figure S1D4, E4). The DMV/1639-infected TOCs then treated with L1-161 (EP4) demonstrated few epithelial cells with deciliation (Figure 5D5), while those infected with Conn A5968 displayed epithelial cell vacuolation (Figure S1E5).

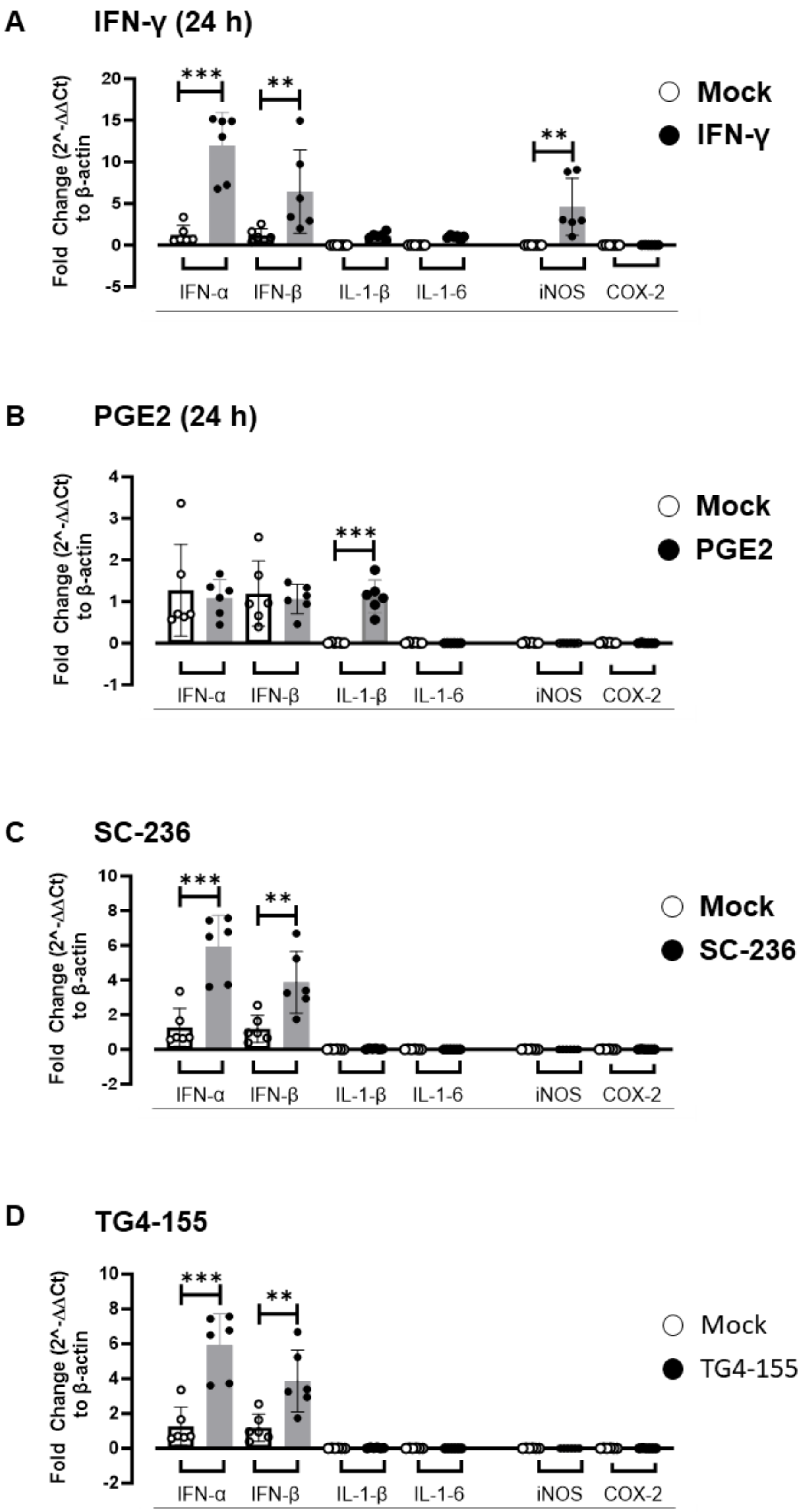
A
IFN-γ (24 h)
Fold Change (2^-ΔΔCt) to β-actin
20
15
10
5
0
-5
***
**
**
IFN-α
IFN-β
IL-1-β
IL-1-6
iNOS
COX-2
Mock
IFN-γ
B
PGE2 (24 h)
Fold Change (2^-ΔΔCt) to β-actin
4
3
2
1
0
-1
***
IFN-α
IFN-β
IL-1-β
IL-1-6
iNOS
COX-2
Mock
PGE2
C
SC-236
Fold Change (2^-ΔΔCt) to β-actin
10
8
6
4
2
0
-2
***
**
IFN-α
IFN-β
IL-1-β
IL-1-6
iNOS
COX-2
Mock
SC-236
D
TG4-155
Fold Change (2^-ΔΔCt) to β-actin
10
8
6
4
2
0
-2
***
**
IFN-α
IFN-β
IL-1-β
IL-1-6
iNOS
COX-2
Mock
TG4-155

E L-161 (24 h)

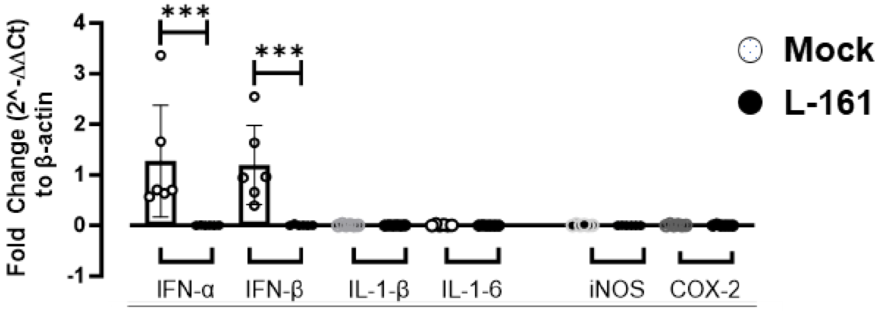

F 420099 (24 h)

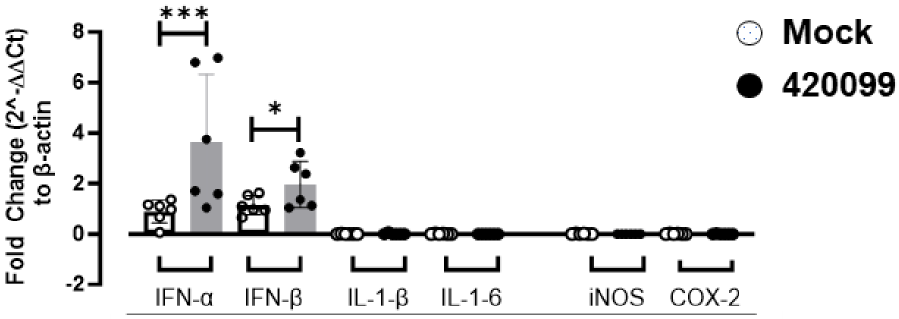

G SP600125 (24 h)

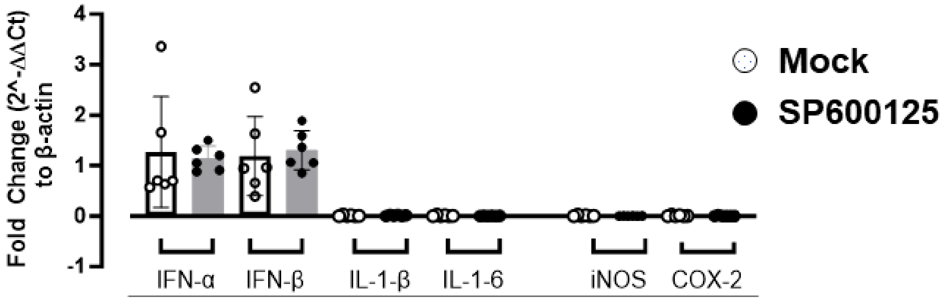

FIG S3 Modulation of Cytokines, iNOS, and COX-2 mRNA Expression in Tracheal Organ Culture Following 24h Treatment with Exogenous IFN-γ, PGE2, and Pharmacological Inhibitors. Quantitative PCR analysis was conducted after RNA extraction and cDNA synthesis from tracheal rings. Tracheal organ cultures were subjected to a 24-hour

treatment with recombinant chicken IFN-γ (100 ng/ml), exogenous PGE2 (10 μg/ml), EP2 and EP4 inhibitors (TG4-155 at 4 nM or L1-61 at 8 nM, respectively), or Janus kinase (JAK) 1 and JAK2 inhibitors (420099 at 15 nM or SP600126 at 40 nM). The presented data, illustrating the mean ± SD from two independent experiments (n = 3/experiment), with all samples run in duplicates, were analyzed using paired Student's t-test for group comparisons. Significant differences (P < 0.05) are denoted by asterisks.

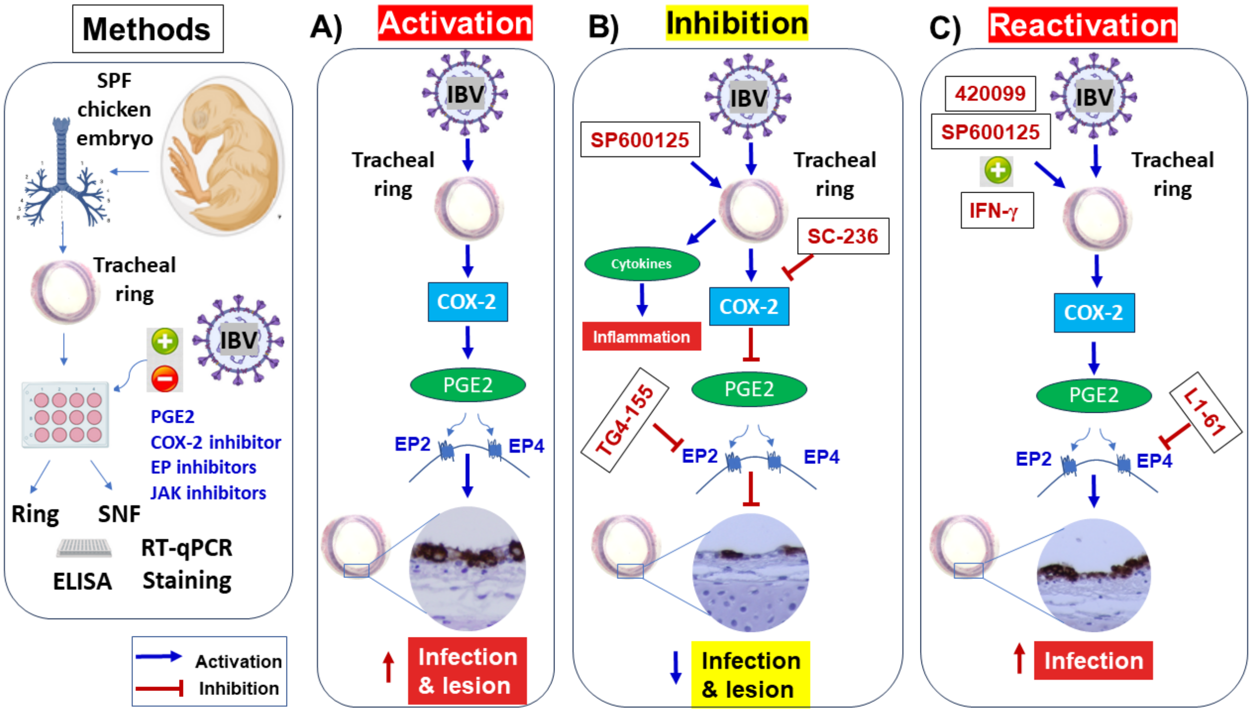

FIG S4 COX-2/PGE2 activation and inhibition in IBV-infected chicken tracheal explants. A 20-day-old chicken embryo yields approximately 20 tracheal rings, which are then exposed to either infection or infection combined with various treatments. (a) Upon IBV infection, upregulation of COX-2 synthesis, leading to increased production and release of PGE-2 and increased infection and lesion of trachea. In addition, exogenous PGE2 treatment exacerbates IBV infection. (b) Pharmacological intervention with IFN-γ or a selective inhibitors to COX-2 (SC-236) , EP2 (TG4-155) and JAK-2 (SP600125) reduces IBV infection and lesion formation. In case of IBV DMV/16 39 was associated with proinflammatory cytokines release and reducing the viral replication. (c) IBV Infection is either enhanced or unaffected by treatment with selective inhibitor (EP4 (L-161) or JAK-1 inhibitors (420099)

or in IFN-γ-pretreated JAK-2 inhibitor (SP600125). The illustrations are simplified from Biorender application.

References

1. Benyeda, Z.; Szeredi, L.; Mató, T.; Süveges, T.; Balka, G.; Abonyi-Tóth, Z.; Rusvai, M.; Palya, V. Comparative histopathology and immunohistochemistry of QX-like, Massachusetts and 793/B serotypes of infectious bronchitis virus infection in chickens. J. Comp. Pathol. 2010, 143, 276–283.
2. Kameka, A. M., et al. (2014). "Induction of innate immune response following infectious bronchitis corona virus infection in the respiratory tract of chickens. Virology 450-451: 114-121.
3. Kamble, N., et al. (2021). "Marek's Disease Virus Modulates T Cell Proliferation via Activation of Cyclooxygenase 2-Dependent Prostaglandin E2." Front Immunol 12: 801781.
4. Villanueva A, I., et al (2011). Synthetic double-stranded RNA oligonucleotides are immunostimulatory for chicken spleen cells. Dev Comp Immunol 35:28–34.
5. Mahmoud ME, Farooq M, Isham IM, Ali A, Hassan MSH, Herath-Mudiyanselage H, Ranaweera HA, Najimudeen SM, Abdul-Careem MF. (2024). Cyclooxygenase-2/prostaglandin E2 pathway regulates infectious bronchitis virus replication in avian macrophages. J Gen Virol 105(1). doi: 10.1099/jgv.0.001949.
6. Xing, Z., & Cardona, C. J. (2009). Preexisting immunity to avian influenza H5N1 in birds. *Emerging Infectious Diseases*, 15(10), 1596-1602.
7. Schijns, V. E. J. C. (2000). Immunological concepts of vaccine adjuvant activity. Current Opinion in Immunology, 12(4), 456-463.
8. Ng, C. T., Fong, L. Y., Abdullah, M. N. H. 2023. Interferon-gamma (IFN-γ): Reviewing its mechanisms and signaling pathways on the regulation of endothelial barrier function. Cytokine, 166, 156208. https://doi.org/10.1016/j.cyto.2023.156208.

9. Alkie TN, Yitbarek A, Hodgins DC, Kulkarni RR, Taha-Abdelaziz K, Sharif S. Development of innate immunity in chicken embryos and newly hatched chicks: a disease control perspective. Avian Pathol. 2019 Aug;48(4):288-310. doi: 10.1080/03079457.2019.1607966. PMID: 31063007.
10. Trinchieri, G. (1995). Interleukin-12: a cytokine at the interface of inflammation and immunity. Advances in Immunology, 70, 83-243. doi:10.1016/S0065-2776(08)60436-6
11. Biswas, S. K., & Mantovani, A. (2010). Macrophage plasticity and interaction with lymphocyte subsets: cancer as a paradigm. Nature Immunology, 11(10), 889-896. doi:10.1038/ni.1937
12. Ng, C. T., et al. (2023). "Interferon-gamma (IFN-γ): Reviewing its mechanisms and signaling pathways on the regulation of endothelial barrier function." Cytokine 166: 156208.
